# Identification of tools used to assess the external validity of randomized controlled trials in reviews: a systematic review of measurement properties

**DOI:** 10.1186/s12874-022-01561-5

**Published:** 2022-04-06

**Authors:** Andres Jung, Julia Balzer, Tobias Braun, Kerstin Luedtke

**Affiliations:** 1 Institute of Health Sciences, Department of Physiotherapy, Pain and Exercise Research Luebeck (P.E.R.L), Universität zu Lübeck, Ratzeburger Allee 160, 23562 Lübeck, Germany; 2 Faculty of Applied Public Health, European University of Applied Sciences, Werftstr. 5, 18057 Rostock, Germany; 3 Division of Physiotherapy, Department of Applied Health Sciences, Hochschule für Gesundheit (University of Applied Sciences), Gesundheitscampus 6‑8, 44801 Bochum, Germany; 4 Department of Health, HSD Hochschule Döpfer (University of Applied Sciences), Waidmarkt 9, 50676 Cologne, Germany

**Keywords:** External validity, Generalizability, Applicability, Measurement properties, Tools, Randomized controlled trial

## Abstract

**Background:**

Internal and external validity are the most relevant components when critically appraising randomized controlled trials (RCTs) for systematic reviews. However, there is no gold standard to assess external validity. This might be related to the heterogeneity of the terminology as well as to unclear evidence of the measurement properties of available tools. The aim of this review was to identify tools to assess the external validity of RCTs. It was further, to evaluate the quality of identified tools and to recommend the use of individual tools to assess the external validity of RCTs in future systematic reviews.

**Methods:**

A two-phase systematic literature search was performed in four databases: PubMed, Scopus, PsycINFO via OVID, and CINAHL via EBSCO. First, tools to assess the external validity of RCTs were identified. Second, studies investigating the measurement properties of these tools were selected. The measurement properties of each included tool were appraised using an adapted version of the COnsensus based Standards for the selection of health Measurement INstruments (COSMIN) guidelines.

**Results:**

38 publications reporting on the development or validation of 28 included tools were included. For 61% (17/28) of the included tools, there was no evidence for measurement properties. For the remaining tools, reliability was the most frequently assessed property. Reliability was judged as “*sufficient*” for three tools (very low certainty of evidence). Content validity was rated as “*sufficient*” for one tool (moderate certainty of evidence).

**Conclusions:**

Based on these results, no available tool can be fully recommended to assess the external validity of RCTs in systematic reviews. Several steps are required to overcome the identified difficulties to either adapt and validate available tools or to develop a better suitable tool.

**Trial registration:**

Prospective registration at Open Science Framework (OSF): 10.17605/OSF.IO/PTG4D.

**Supplementary Information:**

The online version contains supplementary material available at 10.1186/s12874-022-01561-5.

## Background

Systematic reviews are powerful research formats to summarize and synthesize the evidence from primary research in health sciences [1, 2]. In clinical practice, their results are often applied for the development of clinical guidelines and treatment recommendations [3]. Consequently, the methodological quality of systematic reviews is of great importance. In turn, the informative value of systematic reviews depends on the overall quality of the included controlled trials [3, 4]. Accordingly, the evaluation of the internal and external validity is considered a key step in systematic review methodology [4, 5].

Internal validity relates to the systematic error or bias in clinical trials [6] and expresses how methodologically robust the study was conducted. External validity is the inference about the extent to which “a causal relationship holds over variations in persons, settings, treatments and outcomes” [7, 8]. There are plenty of definitions for external validity and a variety of different terms. Hence, external validity, generalizability, applicability, and transferability, among others, are used interchangeably in the literature [9]. Schünemann et al. [10] suggest that: (1) generalizability “may refer to whether or not the evidence can be generalized from the population from which the actual research evidence is obtained to the population for which a healthcare answer is required”; (2) applicability may be interpreted as “whether or not the research evidence answers the healthcare question asked by a clinician or public health practitioner” and (3) transferability is often interpreted as to “whether research evidence can be transferred from one setting to another”. Four essential dimensions are proposed to evaluate the external validity of controlled clinical trials in systematic reviews: patients, treatment (including comparator) variables, settings, and outcome modalities [4, 11]. Its evaluation depends on the specificity of the reviewers´ research question, the review´s inclusion and exclusion criteria compared to the trial´s population, the setting of the study, as well as the quality of reporting these four dimensions.

In health research, however, external validity is often neglected when critically appraising clinical studies [12, 13]. One possible explanation might be the lack of a gold standard for assessing the external validity of clinical trials. Systematic and scoping reviews examined published frameworks and tools for assessing the external validity of clinical trials in health research [9, 12, 14–18]. A substantial heterogeneity of terminology and criteria as well as a lack of guidance on how to assess the external validity of intervention studies was found [9, 12, 15–18]. The results and conclusions of previous reviews were based on descriptive as well as content analysis of frameworks and tools on external validity [9, 14–18]. Although the feasibility of some frameworks and tools was assessed [12], none of the previous reviews evaluated the quality regarding the development and validation processes of the used frameworks and tools.

RCTs are considered the most suitable research design for investigating cause and effect mechanisms of interventions [19]. However, the study design of RCTs is susceptible to a lack of external validity due to the randomization, the use of exclusion criteria and poor willingness of eligible participants to participate [20, 21]. There is evidence that the reliability of external validity evaluations with the same measurement tool differed between randomized and non-randomized trials [22]. In addition, due to differences in requested information from reporting guidelines (e.g. consolidated standards of reporting trials (CONSORT) statement, strengthening the reporting of observational studies in Epidemiology (STROBE) statement), respective items used for assessing the external validity vary between research designs. Acknowledging the importance of RCTs in the medical field, this review focused only on tools developed to assess the external validity of RCTs. The aim was to identify tools to assess the external validity of RCTs in systematic reviews and to evaluate the quality of evidence regarding their measurement properties. Objectives: (1) to identify published measurement tools to assess the external validity of RCTs in systematic reviews; (2) to evaluate the quality of identified tools; (3) to recommend the use of tools to assess the external validity of RCTs in future systematic reviews.

## Methods

This systematic review was reported in accordance with the Preferred Reporting Items for Systematic reviews and Meta-Analyses (PRISMA) 2020 Statement [23] and used an adapted version of the PRISMA flow diagram to illustrate the systematic search strategy used to identify clinimetric papers [24]. This study was conducted according to an adapted version of the COnsensus-based Standards for the selection of health Measurement INstruments (COSMIN) methodology for systematic reviews of measurement instruments in health sciences [25–27] and followed recommendations of the JBI manual for systematic reviews of measurement properties [28]. The COSMIN methodology was chosen since this method is comprehensive and validation processes do not differ substantially between patient-reported outcome measures (PROMs) and measurement instruments of other latent constructs. According to the COSMIN authors, it is acceptable to use this methodology for non-PROMs [26]. Furthermore, because of its flexibility, it has already been used in systematic reviews assessing measurement tools which are not health measurement instruments [29–31]. However, adaptations or modifications may be necessary [26]. The type of measurement instrument of interest for the current study were reviewer-reported measurement tools. Pilot tests and adaptation-processes of the COSMIN methodology are described below (see section “Quality assessment and evidence synthesis”). The definition of each measurement property evaluated in the present review is based on COSMIN´s taxonomy, terminology and definition of measurement properties [32]. The review protocol was prospectively registered on March 6, 2020 in the Open Science Framework (OSF) with the registration DOI: 10.17605/OSF.IO/PTG4D [33].

### Deviations from the preregistered protocol

One of the aims listed in the review protocol was to evaluate the characteristics and restrictions of measurement tools in terms of terminology and criteria for assessing external validity. This issue has been addressed in two recent reviews with a similar scope [9, 17]. Although our eligibility criteria differed, it was concluded that no novel data was available for the present review to extract, since authors of included tools did not describe the definition or construct of interest or cited the same reports. Therefore, this objective was omitted.

### Literature search and screening

A search of the literature was conducted in four databases: PubMed, Scopus, PsycINFO via OVID, and CINAHL via EBSCO. The eligibility criteria and search strategy were predefined in collaboration with a research librarian and is detailed in Table S1 (see Additional file 1). The search strategy was designed according to the COSMIN methodology and consists of the following four key elements: (1) construct (external validity of RCTs from the review authors´perspective), (2) population(s) (RCTs), (3) type of instrument(s) (measurement tools, checklists, surveys etc.), and (4) measurement properties (e.g. validity and reliability) [34]. The four key elements were divided into two main searches (adapted from previous reviews [24, 35, 36]): the phase 1 search contained the first three key elements to identify measurement tools to assess the external validity of RCTs. The phase 2 search aimed to identify studies evaluating the measurement properties of each tool, which was identified and included during phase 1. For this second search, a sensitive PubMed search filter developed by Terwee et al. [37] was applied. Translations of this filter for the remaining databases were taken from the COSMIN website and from other published COSMIN reviews [38, 39] with permission from the authors. Both searches were conducted until March 2021 without restriction regarding the time of publication (databases were searched from inception). In addition, forward citation tracking with Scopus (which is a specialized citation database) was conducted in phase 2 using the ‘cited by’-function. The Scopus search filter was then entered into the ‘search within results’-function. The results from the forward citation tracking with Scopus were added to the database search results into the Rayyan app for screening. Reference lists of the retrieved full-text articles and forward citations with PubMed were scanned manually for any additional studies by one reviewer (AJ) and checked by a second reviewer (KL).

Title and abstract screening for both searches and the full-text screening during phase 2 were performed independently by at least two out of three involved researchers (AJ, KL & TB). For pragmatic reasons, full-text screening and tool/data extraction in phase 1 was performed by one reviewer (AJ) and checked by a second reviewer (TB). This screening method is acceptable for full-text screening as well as data extraction [40]. Data extraction for both searches was performed with a predesigned extraction sheet based on the recommendations of the COSMIN user manual [34]. The Rayyan Qatar Computing Research Institute (QCRI) web app [41] was used to facilitate the screening process (both searches) according to a priori defined eligibility criteria. A pilot test was conducted for both searches in order to reach agreement between the reviewers during the screening process. For this purpose, the first 100 records in phase 1 and the first 50 records in phase 2 (sorted by date) in the Rayyan app were screened by two reviewers independently and subsequently, issues regarding the feasibility of screening methods were discussed in a meeting.

### Eligibility criteria

#### Phase 1 search (identification of tools)

Records were considered for inclusion based on their title and abstract according to the following criteria: (1) records that described the development and or implementation (application), e.g. manual or handbook, of any tool to assess the external validity of RCTs; (2) systematic reviews that applied tools to assess the external validity of RCTs and which explicitly mentioned the tool in the title or abstract; (3) systematic reviews or any other publication potentially using a tool for external validity assessment, but the tool was not explicitly mentioned in the title or abstract; (4) records that gave other references to, or dealt with, tools for the assessment of external validity of RCTs, e.g. method papers, commentaries.

The full-text screening was performed to extract or to find references to potential tools. If a tool was cited, but not presented or available in the full-text version, the internet was searched for websites on which this tool was presented, to extract and review for inclusion. Potential tools were extracted and screened for eligibility as follows: measurement tools aiming to assess the external validity of RCTs and designed for implementation in systematic reviews of intervention studies. Since the terms external validity, applicability, generalizability, relevance and transferability are used interchangeably in the literature [10, 11], tools aiming to assess one of these constructs were eligible. Exclusion criteria: (1) The multidimensional tool included at least one item related to external validity, but it was not possible to assess and interpret external validity separately. (2) The tool was developed exclusively for study designs other than RCTs. (3) The tool contained items assessing information not requested in the CONSORT-Statement [42] (e.g. cost-effectiveness of the intervention, salary of health care provider) and these items could not be separated from items on external validity. (4) The tool was published in a language other than English or German. (5) The tool was explicitly designed for a specific medical profession or field and cannot be used in other medical fields.

#### Phase 2 search (identification of reports on the measurement properties of included tools)

For the phase 2 search, records evaluating the measurement properties of at least one of the included measurement tools were selected. Reports only using the measurement tool as an outcome measure without the evaluation of at least one measurement property were excluded. If a report did not evaluate the measurement properties of a tool, it was also excluded. Hence, reports providing data on the validity or the reliability of sum-scores of multidimensional tools, only, were excluded if the dimension “external validity” was not evaluated separately.

If there was missing data or information (phase 1 or phase 2), the corresponding authors were contacted.

### Quality assessment and evidence synthesis

All included reports were systematically evaluated: (1) for their methodological quality by using the adapted COSMIN Risk of Bias (RoB) checklist [25] and (2) against the updated criteria for good measurement properties [26, 27]. Subsequently, all available evidence for each measurement property for the individual tool were summarized and rated against the updated criteria for good measurement properties and graded for their certainty of evidence, according to COSMIN´s modified GRADE approach [26, 27]. The quality assessment was performed by two independent reviewers (AJ & JB). In case of irreconcilable disagreement, a third reviewer (TB) was consulted to reach consensus.

The COSMIN RoB checklist is a tool [25, 27, 32, 43] designed for the systematic evaluation of the methodological quality of studies assessing the measurement properties of health measurement instruments [25]. Although this checklist was specifically developed for systematic reviews of PROMs, it can also be used for reviews of non-PROMs [26] or measurement tools of other latent constructs [28, 29]. As mentioned in the COSMIN user manual, adaptations for some items in the COSMIN RoB checklist might be necessary, in relation to the construct being measured [34]. Therefore, pilot tests were performed for the assessment of measurement properties of tools assessing the quality of RCTs before data extraction, aiming to ensure feasibility during the planned evaluation of the included tools. The pilot tests were performed with a random sample of publications on measurement instruments of potentially relevant tools. After each pilot test, results and problems regarding the comprehensibility, relevance and feasibility of the instructions, items, and response options in relation to the construct of interest were discussed. Where necessary, adaptations and/or supplements were added to the instructions of the evaluation with the COSMIN RoB checklist. Saturation was reached after two rounds of pilot testing. Substantial adaptations or supplements were required for Box 1 (‘development process’) and Box 10 (‘responsiveness’) of the COSMIN RoB checklist. Minor adaptations were necessary for the remaining boxes. The specification list, including the adaptations, can be seen in Table S2 (see Additional file 2). The methodological quality of included studies was rated via the four-point rating scale of the COSMIN RoB checklist as “inadequate”, “doubtful”, “adequate”, or “very good” [25]. The lowest score of any item in a box is taken to determine the overall rating of the methodological quality of each single study on a measurement property [25].

After the RoB-assessment, the result of each single study on a measurement property was rated against the updated criteria for good measurement properties for content validity [27] and for the remaining measurement properties [26] as “sufficient” (+), “insufficient” (-), or “indeterminate” (?). These ratings were summarized and an overall rating for each measurement property was given as “sufficient” (+), “insufficient” (-), “inconsistent” (±), or “indeterminate” (?). However, the overall rating criteria for good content validity was adapted to the research topic of the present review. This method usually requires an additional subjective judgement from reviewers [44]. Since one of the biggest limitations within this field of research is the lack of consensus on terminology and criteria as well as on how to assess the external validity [9, 12], a reviewers’ subjective judgement was considered inappropriate. After this issue was also discussed with one leading member of the COSMIN steering committee, the reviewers’ rating was omitted. A “sufficient” (+) overall rating was given if there was evidence of face or content validity of the final version of the measurement tool assessed by a user or expert panel. Otherwise, the rating “indeterminate” (?) or “insufficient” (-) was used for the content validity.

The summarized evidence for each measurement property for the individual tool was graded using COSMIN´s modified GRADE approach [26, 27]. The certainty (quality) of evidence was graded as “high”, “moderate”, “low”, or “very low” according to the approach for content validity [27] and for the remaining measurement properties [26]. COSMIN´s modified GRADE approach distinguishes between four factors influencing the certainty of evidence: risk of bias, inconsistency, indirectness, and imprecision. The starting point for all measurement properties is high certainty of evidence and is subsequently downgraded by one to three levels per factor when there is risk of bias, (unexplained) inconsistency, imprecision (not considered for content validity [27]), or indirect results [26, 27]. If there is no study on the content validity of a tool, the starting point for this measurement property is “moderate” and is subsequently downgraded depending on the quality of the development process [27]. The grading process according to COSMIN [26, 27] is described in Table S4. Selective reporting bias or publication bias is not taken into account in COSMIN´s modified GRADE approach, because of a lack of registries for studies on measurement properties [26].

The evidence synthesis was performed qualitatively according to the COSMIN methodology [26]. If several reports revealed homogenous quantitative data (e.g. same statistics, population) on internal consistency, reliability, measurement error or hypotheses testing of a measurement tool, pooling the results was considered using generic inverse variance (random effects) methodology and weighted means as well as 95% confidence intervals for each measurement property [34]. No subgroup analysis was planned. However, statistical pooling was not possible in the present review.

We used three criteria for the recommendation of a measurement tool in accordance with the COSMIN manual: (A) “Evidence for sufficient content validity (any level) and at least low-quality evidence for sufficient internal consistency” for a tool to be recommended; (B) tool “categorized not in A or C” and further research on the quality of this tool is required to be recommended; and (C) tool with “high quality evidence for an insufficient psychometric property” and this tool should not be recommended [26].

## Results

### Literature search and selection process

**Fig. 1:**
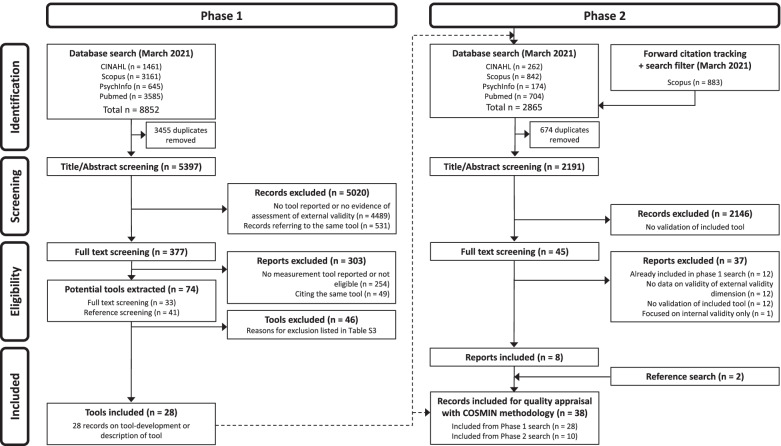
Flow diagram “of systematic search strategy used to identify clinimetric papers”[24]

Figure 1 shows the selection process. In the phase 1 search, from 5397 non-duplicate records, 5020 irrelevant records were excluded. 377 reports were screened, and 74 potential tools were extracted. After reaching consensus, 46 tools were excluded (reasons for exclusion are presented in Table S3 (see Additional file 3)) and finally 28 were included. Any disagreements during the screening process were resolved through discussion. There was one case during the full-text screening process in the phase 1 search, in which the whole review team was involved to reach consensus about the inclusion/exclusion of two tools (Agency for Healthcare Research and Quality (AHRQ) criteria for applicability and TRANSFER approach, both listed in Table S3).

In the phase 2 search, 2191 non-duplicate records were screened for title and abstract. 2146 records were excluded as they did not assess any measurement property of the included tools. Of 45 reports, 8 reports were included. The most common reason for exclusion was that reports evaluating the measurement properties of multidimensional tools did not evaluate external validity as a separate dimension. For example, one study assessing the interrater reliability of the GRADE method [45] was identified during full-text screening, but had to be excluded, since it did not provide separate data on the reliability of the indirectness domain (representing external validity). Two additional reports were included during reference screening. Any disagreements during the screening process were resolved through discussion.

Thirty-eight publications on the development or evaluation of the measurement properties of 28 included tools were included for quality appraisal according to the adapted COSMIN guidelines.

We contacted the corresponding authors of three reports [46–48] for additional information. One corresponding author did reply [48].

### Methods to assess the external validity of RCTs

During full-text screening in phase 1, several concepts to assess the external validity of RCTs were found (Table 1). Two main concepts were identified: experimental/statistical methods and non-experimental methods. The experimental/statistical methods were summarized and collated into five subcategories giving a descriptive overview of the different approaches used to assess the external validity. However, according to our eligibility criteria, these methods were excluded, since they were not developed for the use in systematic reviews of interventions. In addition, a comparison of these methods as well as appraisal of risk of bias with the COSMIN RoB checklist would not have been feasible. Therefore, the experimental/statistical methods described below were not included for further evaluation.

**Table 1:** Experimental/statistical methods to evaluate the EV of RCTs

**1. **Comparing differences of characteristics and/or NNT analysis from not-enrolled eligible patients with enrolled patients [49–52]
**2**. Conduction of observational studies to assess the “real world” applicability of RCTs [20, 53, 54]
**3**. Meta-analysis of patient characteristics data from RCTs [55, 56]
**4**. Comparison of data from RCTs with data from health record database and/or other epidemiological data: **a)** retrospectively [55–59] **b)** simulation-based (a priori and retrospective) [60, 61]
**5**. Review of exclusion criteria in RCTs which would limit the EV [62]

*Abbreviations: EV *external validity, *NNT *numbers needed to treat, *RCT *randomized controlled trial

For non-experimental methods, please refer to Table 2

#### Characteristics of included measurement tools

The included tools and their characteristics are listed in Table 2. Overall, the tools were heterogenous with respect to the number of items or dimensions, response options and development processes. The number of items varied between one and 26 items and the response options varied between 2-point-scales to 5-point-scales. Most tools used a 3-point-scale (*n* = 20/28, 71%). For 14/28 (50%) of the tools, the development was not described in detail [63–76]. Seven review authors appear to have developed their own tool but did not provide any information on the development process [63–68, 71].

**Table 2:** Characteristics of included tools

Dimension and/or tool	Authors	Construct(s), as described by the authors	Target population	Domains, nr. of items	Response options	Development and validation
“Applicability”-dimension of LEGEND	Clark et al. [77]	Applicability of results to treating patients	P1: RCTs and CCTs P2: reviewers and clinicians	3 items	3-point-scale	Deductive and inductive item-generation. Tool was pilot tested among an interprofessional group of clinicians.
“Applicability”-dimension of Carr´s evidence-grading scheme	Carr et al. [63]	Generalizability of study population	P1: clinical trials P:2 authors of SRs	1 item	3-point-classification-scale	No specific information on tool development.
Bornhöft´s checklist	Bornhöft et al. [78]	External validity (EV) and Model validity (MV) of clinical trials	P1: clinical trials P2: authors of SRs	4 domains with 26 items for EV and MV each	4-point-scale	Development with a comprehensive, deductive item-generation from the literature. Pilot-tests were performed, but not for the whole scales.
Cleggs´s external validity assessment	Clegg et al. [64]	Generalizability of clinical trials to England and Wales	P1: clinical trials P2: authors of SRs and HTAs	5 items	3-point-scale	No specific information on tool development
Clinical applicability	Haraldsson et al. [66]	Report quality and applicability of intervention, study population and outcomes	P1: RCTs P2: reviewers	6 items	3-point-scale and 4-point-scale	No specific information on tool development
Clinical Relevance Instrument	Cho & Bero [79]	Ethics and Generalizability of outcomes, subjects, treatment and side effects	P1: clinical trials P2: reviewers	7 items	3-point-scale	Tool was pilot tested on 10 drug studies. Content validity was confirmed by 7 reviewers with research experience. - interrater reliability: ICC = 0.56 (*n* = 127) [80]
“Clinical Relevance” according to the CCBRG	Van Tulder et al. [81]	Applicability of patients, interventions and outcomes	P1: RCTs P2: authors of SRs	5 items	3-point-scale (Staal et al., 2008)	Deductive item-generation for Clinical Relevance. Results were discussed in a workshop. After two rounds, a final draft was circulated for comments among editors of the CCBRG.
Clinical Relevance Score	Karjalainen et al. [68]	Report quality and applicability of results	P1: RCTs P2: reviewers	3 items	3-point-scale	No specific information on tool development.
Estrada´s applicability assessment criteria	Estrada et al. [82]	Applicability of population, intervention, implementation and environmental context to Latin America	P1: RCTs P2: reviewers	5 domains with 8 items	3-point-scale for each domain	Deductive item generation from the review by Munthe-Kaas et al. [17]. Factors and items were adapted, and pilot tested by the review team (*n* = 4) until consensus was reached.
EVAT (External Validity Assessment Tool)	Khorsan & Crawford [83]	External validity of participants, intervention, and setting	P1: RCTs and non-randomized studies P2: reviewers	3 items	3-point-scale	Deductive item-generation. Tool developed based on the GAP-checklist [76] and the Downs and Black-checklist [22]. Feasibility was tested and a rulebook was developed but not published.
“External validity”-dimension of the Downs & Black-Checklist	Downs & Black [22]	Representativeness of study participants, treatments and settings to source population or setting	P1: RCTs and non-randomised studies P2: reviewers	3 items	3-point-scale	Deductive item-generation, pilot test and content validation of pilot version. Final version tested for: - internal consistency: KR-20 = 0.54 (*n* = 20), - reliability: test-retest: *k* = -0.05-0.48 and 10–15% disagreement (measurement error) (*n* = 20), [22] interrater reliability: *k* = -0.08-0.00 and 5–20% disagreement (measurement error) (*n* = 20) [22]; ICC = 0.76 (*n* = 20) [84]
“External validity”-dimension of Foy´s quality checklist	Foy et al. [65]	External validity of patients, settings, intervention and outcomes	P1: intervention studies P2: reviewers	6 items	not clearly described	Deductive item-generation. No further information on tool development.
“External validity”-dimension of Liberati´s quality assessment criterias	Liberati et al. [69]	Report quality and generalizability	P1: RCTS P2: reviewers	9 items	dichotomous and 3-point-scale	Tool is a modified version of a previously developed checklist [85] with additional inductive item-generation. No further information on tool development.
“External validity”-dimension of Sorg´s checklist	Sorg et al. [71]	External validity of population, interventions, and endpoints	P1: RCTs P2: reviewers	4 domains with 11 items	not clearly described	Developed based on Bornhöft et al. [78] No further information on tool development.
“external validity”-criteria of the USPSTF	USPSTF Procedure manual [73]	Generalizability of study population, setting and providers for US primary care	P1: clinical studies P2: USPSTF reviewers	3 items	Sum-score- rating: 3-point-scale	Tool developed for USPSTF reviews. No specific information on tool development. - interrater reliability: ICC = 0.84 (*n* = 20) [84]
FAME (Feasibility, Appropriateness, Meaningfulness and Effectiveness) scale	Averis et al. [70]	Grading of recommendation for applicability and ethics of intervention	P1: intervention studies P2: reviewers	4 items	5-point-scale	The FAME framework was created by a national group of nursing research experts. Deductive and inductive item-generation. No further information on tool development.
GAP (Generalizability, Applicability and Predictability) checklist	Fernandez-Hermida et al. [76]	External validity of population, setting, intervention and endpoints	P1: RCTs P2: Reviewers	3 items	3-point-scale	No specific information on tool development.
Gartlehner´s tool	Gartlehner et al. [86]	To distinguish between effectiveness and efficacy trials	P1: RCTs P2: reviewers	7 items	Dichotomous	Deductive and inductive item-generation. - criterion validity testing with studies selected by 12 experts as gold standard.: specificity = 0.83, sensitivity = 0.72 (*n* = 24) - measurement error: 78.3% agreement (*n* = 24) - interrater reliability: *k* = 0.42 (*n* = 24) [86]; *k* = 0.11–0.81 (*n* = 151) [87]
Green & Glasgow´s external validity quality rating criteria	Green & Glasgow [88]	Report quality for generalizability	P1: trials (not explicitly described) P2: reviewers	4 Domains with 16 items	Dichotomous	Deductive item-generation. Mainly based on the Re-Aim framework.[89] - interrater reliability: ICC = 0.86 (*n* = 14) [90] - discriminative validity: TREND studies report on 77% and non-TREND studies report on 54% of scale items (*n* = 14) [90] - ratings across included studies (*n* = 31) [91], no hypothesis was defined
“Indirecntess”-dimension of the GRADE handbook	Schünemann et al. [92]	Differences of population, interventions, and outcome measures to research question	P1: intervention studies P2: authors of SRs, clinical guidelines and HTAs	4 items	Overall: 3-point-scale (downgrading options)	Deductive and inductive item-generation, pilot-testing with 17 reviewers (*n* = 12) [48]. - interrater reliability: ICC = 0.00–0.13 (*n* > 100) [93]
Loyka´s external validity framework	Loyka et al. [75]	Report quality for generalizability of research in psychological science	P1: intervention studies P2: researchers	4 domains with 15 items	Dichotomous	Deductive item generation (including Green & Glasgow [88]) and adaptation for psychological science. No further information on tool development. - measurement error: 60-100% agreement (*n* = 143)
Modified “Indirectness” of the Checklist for GRADE	Meader et al. [94]	Differences of population, interventions, and outcome measures to research question.	P1: meta-analysis of RCTs P2: authors of SRs, clinical guidelines and HTAs	5 items	Item-level: 2-and 3-point-scale Overall: 3-point-scale (grading options)	Developed based on GRADE method, two phase pilot-tests, - interrater reliability: kappa was poor to almost perfect on item-level [94] and *k* = 0.69 for overall rating of indirectness (*n* = 29) [95]
external validity checklist of the NHMRC handbook	NHMRC handbook [74]	external validity of an economic study	P1: clinical studies P2: clinical guideline developers, reviewers	6 items	3-point-scale	No specific information on tool development.
revised GATE in NICE manual (2012)	NICE manual [72]	Generalizability of population, interventions and outcomes	P1: intervention studies P2: reviewers	2 domains with 4 items	3-point-scale and 5-point-scale	Based on Jackson et al. [96] No specific information on tool development.
RITES (Rating of Included Trials on the Efficacy-Effectiveness Spectrum)	Wieland et al. [47]	To characterize RCTs on an efficacy-effectiveness continuum.	P1: RCTs P2: reviewers	4 items	5-point-likert-scale	Deductive and inductive item-generation, modified Delphi procedure with 69–72 experts, pilot testing in 4 Cochrane reviews, content validation with Delphi procedure and core expert group (*n* = 14) [47], - interrater reliability: ICC = 0.54-1.0 (*n* = 22) [97] - convergent validity with PRECIS 2 tool: *r *= 0.55 correlation (*n* = 59) [97]
Section A (Selection Bias) of EPHPP (Effective Public health Practice Project) tool	Thomas et al. [98]	Representativeness of population and participation rate.	P1: clinical trials P2: reviewers	2 items	Item-level: 4-point-scale and 5-point-scale Overall: 3-point-scale	Deductive item-generation, pilot-tests, content validation by 6 experts, - convergent validity with Guide to Community Services (GCPS) instrument: 52.5–87.5% agreement (*n* = 70) [98] - test-retest reliability: *k* = 0.61–0.74 (*n* = 70) [98] *k* = 0.60 (*n* = 20) [99]
Section D of the CASP checklist for RCTs	CASP Programme [100]	Applicability to local population and outcomes	P1: RCTs P2: participants of workshops, reviewers	2 items	3-point-scale	Deductive item-generation, development and pilot-tests with group of experts.
Whole Systems research considerations´ checklist	Hawk et al. [67]	Applicability of results to usual practice	P1: RCTs P2: Reviewers (developed for review)	7 domains with 13 items	Item-level: dichotomous Overall: 3-point-scale	Deductive item-generation. No specific information on tool development.

*Abbreviations: CASP *Critical Appraisal Skills Programme, *CCBRG *Cochrane Collaboration Back Review Group, *CCT *controlled clinical trial, *GATE *Graphical Appraisal Tool for Epidemiological Studies, *GRADE *Grading of Recommendations Assessment, Development and Evaluation, *HTA *Health Technology Assessment, *ICC *intraclass correlation, *LEGEND *Let Evidence Guide Every New Decision, *NICE *National Institute for Health and Care Excellence, *PRECIS *PRagmatic Explanatory Continuum Indicator Summary, *RCT *randomized controlled trial, *TREND*  Transparent Reporting of Evaluations with Nonrandomized Designs, *USPSTF *U.S. Preventive Services Task Force

The constructs aimed to be measured by the tools or dimensions of interest are diverse. Two of the tools focused on the characterization of RCTs on an efficacy-effectiveness continuum [47, 86], three tools focused predominantly on the report quality of factors essential to external validity [69, 75, 88] (rather than the external validity itself), 18 tools aimed to assess the representativeness, generalizability or applicability of population, setting, intervention, and/or outcome measure to usual practice [22, 63–65, 70, 71, 73, 74, 76–78, 81–83, 92, 94, 100], and five tools seemed to measure a mixture of these different constructs related to external validity [66, 68, 72, 79, 98]. However, the construct of interest of most tools was not described adequately (see below).

### Measurement properties

The results of the methodological quality assessment according to the adapted COSMIN RoB checklist are detailed in Table 3. If all data on hypotheses testing in an article had the same methodological quality rating, they were combined and summarized in Table 3 in accordance with the COSMIN manual [34]. The results of the ratings against the updated criteria for good measurement properties and the overall certainty of evidence, according to the modified GRADE approach, can be seen in Table 4. The detailed grading is described in Table S4 (see Additional file 4). Disagreements between reviewers during the quality assessment were resolved through discussion.

**Table 3:** Methodological quality of included studies based on COSMIN risk of bias (RoB) checklist

Tool or dimension	Report	Content validity				Internal structure			Remaining measurement properties			
		**Development**	**2.1** CB	**2.2** RE	**2.3** CH	**Structural validity**	**Internal consistency**	**Cross-cultural validity**	**Reliability**	**Measurement error**	**Criterion validity**	**Construct validity**
**“Applicability”-dimension of LEGEND**	Clark et al. [77]	doubtful										
**“Applicability”-dimension of Carr´s evidence-grading scheme**	Carr et al. [63]	inadequate										
**Bornhöft´s checklist**	Bornhöft et al. [78]	inadequate										
**Cleggs´s external validity assessment**	Clegg et al. [64]	inadequate										
**Clinical Applicability**	Haraldsson et al. [66]	inadequate										
**Clinical Relevance Instrument**	Cho & Bero [79]	doubtful	doubtful	doubtful	doubtful							
	Cho & Bero [80]								adequate			
**Clinical Relevance according to the CCBRG**	Van Tulder et al. [81]	inadequate	doubtful	doubtful	doubtful							
**Clinical relevance scores (Karjalainen´s)**	Karjalainen et al. [68]	inadequate										
**Estrada´s applicability assessment criteria**	Estrada et al. [82]	doubtful										
**EVAT**	Khorsan & Crawford [83]	doubtful										
**“External validity”-dimension of the Downs & Black Checklist**	Downs & Black [22]	doubtful	doubtful	doubtful	doubtful		doubtful		very good^a^	inadequate^a^		adequate
									very good^a^	inadequate^a^		
	O´Connor et al. [84]								very good			
**“External validity”-dimension of Foy´s quality checklist**	Foy et al. [65]	inadequate										
**“External validity”-dimension of Liberati´s quality assessment criteria**	Liberati et al. [69]	inadequate										
**“External validity”-dimension of Sorg´s checklist**	Sorg et al. [71]	inadequate										
**“External validity”-criteria of the USPSTF**	USPSTF manual [73]	inadequate										
	O´Connor et al. [84]								very good			
**FAME scale**	Averis et al. [70]	inadequate										
**GAP checklist**	Fernandez-Hermida et al. [76]	inadequate										
**Gartlehner´s tool**	Gartlehner et al. [86]	inadequate							very good	adequate	adequate	
	Zettler et al. [87]								very good			
**Green & Glasgow´s external validity quality rating criteria**	Green & Glasgow [88]	inadequate										
	Laws et al. [91]											doubtful
	Mirza et al. [90]								adequate			doubtful
**“Indirecntess”-dimension from the GRADE Handbook **[92]	Atkins et al. [48]	adequate										
	Wu et al. [93]								inadequate			
**Loyka´s external validity framework**	Loyka et al.^75^	doubtful								adequate		
**modified “Indirectness” of the Checklist for GRADE**	Meader et al. [94]	adequate							adequate^b^			
	Llewellyn et al. [95]											
**External validity checklist of the NHMRC Handbook**	NHMRMC Handbook [74]	inadequate										
**revised GATE in the NICE manual**	NICE Guideline [72]	inadequate										
**RITES tool**	Wieland et al. [47]	adequate	adequate	very good	very good							
	Aves et al. [97, 101]								inadequate			very good
**“Selection Bias”-dimension (Section A) of the EPHPP tool**	Thomas et al. [98]	inadequate	doubtful	doubtful	doubtful				doubtful			doubtful
	Armijo-Olivo et al. [99]								doubtful			
**Section D of the CASP checklist for RCTs**	Critical Appraisal Skills Programme [100]	inadequate										
**Whole Systems research considerations´checklist**	Hawk et al. [67]	inadequate										

Fields left blank indicate that those measurement properties were not assessed by the study authors

*Abbreviations: CB *comprehensibility, *RE *relevance, *CV *comprehensiveness, *CCBRG *Cochrane Collaboration Back Review Group, *EPHPP *Effective Public Health Practice Project, *EVAT *External Validity Assessment Tool, *FAME *Feasibility, Appropriateness, Meaningfulness and Effectiveness, *GAP *Generalizability, Applicability and Predictability; *GATE* Graphical Appraisal Tool for Epidemiological Studies, *GRADE *Grading of Recommendations Assessment, Development and Evaluation; *LEGEND* Let Evidence Guide Every New Decision, *NHMRC *National Health & Medical Research Council, *NICE *National Institute for Health and Care Excellence, *RITES* Rating of Included Trials on the Efficacy-Effectiveness Spectrum, *USPSTF *U.S. Preventive Services Task Force

^a^ two studies on reliability (test-retest & inter-rater reliability) in the same article

^b^ results from the same study on reliability reported in two articles [94, 95]

**Table 4:** Criteria for good measurement properties & certainty of evidence according to the modified GRADE method

Tool or dimension	Content validity	Internal consistency	Reliability	Measurement error	Criterion validity	Construct validity
**“Applicability”-dimension of LEGEND** [77]						
CGMP	(?)					
GRADE	Low					
**“Applicability”-dimension of Carr´s evidence-grading scheme** [63]						
CGMP	(?)					
GRADE	Very Low					
**Bornhöft´s checklist** [78]						
CGMP	(?)					
GRADE	Very Low					
**Cleggs´s external validity assessment** [64]						
CGMP	(?)					
GRADE	Very Low					
**Clinical Applicability** [66]						
CGMP	(?)					
GRADE	Very Low					
**Clinical Relevance Instrument** [79, 80]						
CGMP	(?)		(-)			
GRADE	Moderate		Moderate			
**Clinical Relevance according to the CCBRG** [81]						
CGMP	(?)					
GRADE	Moderate					
**Clinical relevance scores** [68]						
CGMP	(?)					
GRADE	Very Low					
**Estrada´s applicability assessment criteria** [82]						
CGMP	(?)					
GRADE	Very Low					
**External Validity Assessment Tool (EVAT)** [83]						
CGMP	(?)					
GRADE	Low					
**“External validity”-dimension of the Downs & Black Checklist** [22, 84]						
CGMP	(?)	(?)	(±)^a^	(?)		(-)
GRADE	Moderate	Very Low	Moderate	Very Low		Very Low
**“External validity”-dimension of Foy´s quality checklist** [65]						
CGMP	(?)					
GRADE	Very Low					
**“External validity”-dimension of Liberati´s quality assessment criteria** [69]						
CGMP	(?)					
GRADE	Very Low					
**“External validity”-dimension of Sorg´s checklist** [71]						
CGMP	(?)					
GRADE	Very Low					
**“External validity”-criteria of the USPSTF manual** [73, 84]						
CGMP	(?)		(+)			
GRADE	Very Low		Very Low			
**Feasibility, Appropriateness, Meaningfulness and Effectiveness (FAME) scale** [70]						
CGMP	(?)					
GRADE	Very Low					
**Generalizability, Applicability and Predictability (GAP) checklist** [76]						
CGMP	(?)					
GRADE	Very Low					
**Gartlehner´s tool** [86, 87]						
CGMP	(?)		(-)	(?)	(+)	
GRADE	Very Low		Moderate	Very Low	Very Low	
**Green & Glasgow´s external validity quality rating criteria** [88, 90, 91]						
CGMP	(?)		(+)			(-)
GRADE	Very Low		Very Low			Very Low
**“Indirecntess”-dimension from the GRADE Handbook** [48, 92, 93]						
CGMP	(?)		(-)			
GRADE	Moderate		Very Low			
**Loyka´s external validity framework** [75]						
CGMP	(?)			(?)		
GRADE	Very Low			Low		
**modified “Indirectness” of the Checklist for GRADE** [94, 95]						
CGMP	(?)		(-)			
GRADE	Low		Very Low			
**External validity checklist of the National Health & Medical Research Council (NHMRC) Handbook** [74]						
CGMP	(?)					
GRADE	Very Low					
**revised Graphical Appraisal Tool for Epidemiological Studies (GATE)** [72]						
CGMP	(?)					
GRADE	Very Low					
**Rating of Included Trials on the Efficacy-Effectiveness Spectrum (RITES)** [47, 97]						
CGMP	(+)		(+)			(+)
GRADE	Moderate		Very Low			Low
**“Selection Bias”-dimension (Section A) of EPHPP** [98, 99]						
CGMP	(?)		(-)			(+)
GRADE	Moderate		Low			Very Low
**Section C of the CASP checklist for RCTs** [100]						
CGMP	(?)					
GRADE	Very Low					
**Whole Systems research considerations´checklist** [67]						
CGMP	(?)					
GRADE	Very Low					

*Abbreviations: CCBRG *Cochrane Collaboration Back Review Group, *CGMP *criteria for good measurement properties, *EPHPP *Effective Public Health Practice Project, *GRADE *Grading of Recommendations Assessment, Development and Evaluation, *LEGEND *Let Evidence Guide Every New Decision, *NICE *National Institute for Health and Care Excellence, *USPSTF *U.S. Preventive Services Task Force;

Criteria for good measurement properties: (+) = sufficient; (?) = indeterminate; (-) = insufficient, (±) or inconsistent

Level of evidence according to the modified GRADE approach: high, moderate, low, or very low evidence.Note: the measurement properties “structural validity” and “cross-cultural validity” are not presented in this table, since they were not assessed in any of the included studies

Fields left blank indicate that those measurement properties were not assessed by the study authors

^a^ please refer to Table S4 for more information on reliability of the “external validity”-dimension of the Downs & Black checklist

#### Content validity

The methodological quality of the development process was “inadequate” for 19/28 (68%) of the included tools [63–66, 68–74, 76, 78, 81, 88, 98, 100]. This was mainly due to insufficient description of the construct to be measured, the target population, or missing pilot tests. Six development studies had a “doubtful” methodological quality [22, 75, 77, 79, 82, 83] and three had an “adequate” methodological quality [47, 48, 94].

There was evidence for content validation of five tools [22, 47, 79, 81, 98]. However, the methodological quality of the content validity studies was “adequate” and “very good” only for the Rating of Included Trials on the Efficacy-Effectiveness Spectrum (RITES) tool [47] and “doubtful” for Cho´s Clinical Relevance Instrument [79], the “external validity”-dimension of the Downs & Black-checklist [22], the “Selection Bias”-dimension of the Effective Public Health Practice Project (EPHPP) tool [98], and the “Clinical Relevance” tool [81]. The overall certainty of evidence for content validity was “very low” for 19 tools (mainly due to very serious risk of bias and serious indirectness) [63–76, 78, 82, 86, 88, 100], “low” for three tools (mainly due to serious risk of bias or serious indirectness) [77, 83, 94] and “moderate” for six tools (mainly due to serious risk of bias or serious indirectness) [22, 47, 79, 81, 92, 98]. All but one tool had an “indeterminate” content validity. The RITES tool [47] had “moderate” certainty of evidence for “sufficient” content validity.

#### Internal consistency

One study assessed the internal consistency for one tool (“external validity”-dimension of the Downs & Black-checklist) [22]. The methodological quality of this study was “doubtful” due to a lack of evidence on unidimensionality (or structural validity). Thus, this tool had a “very low” certainty of evidence for “indeterminate” internal consistency. Reasons for downgrading were a very serious risk of bias and imprecision.

#### Reliability

Out of 13 studies assessing the reliability of 9 tools, eleven evaluated the interrater reliability [80, 84, 86, 87, 90, 93–95, 97, 99], one the test-retest reliability [98], and one evaluated both [22]. Two studies had an “inadequate” [93, 101], two had a “doubtful” [98, 99], three had an “adequate” [80, 91, 94, 95], and six had a “very good” methodological quality [22, 84, 86, 87]. The overall certainty of evidence was “very low” for five tools (reasons for downgrading please refer to Table S4) [47, 73, 88, 92, 94]. The certainty of evidence was “low” for the “Selection Bias”-dimension of the EPHPP tool (due to serious risk of bias and imprecision) [98] and “moderate” for Gartlehner´s tool [86], the “external validity”-dimension of the Downs & Black-checklist [22], as well as the clinical relevance instrument [79] (due to serious risk of bias and indirectness).

Out of nine evaluated tools, the Downs & Black-checklist [22] had “inconsistent” results on reliability. The Clinical Relevance Instrument [79], Gartlehner´s tool [86], the “Selection Bias”-dimension of the EPHPP [98], the indirectness-dimension of the GRADE handbook [92] and the modified indirectness-checklist [94] had an “insufficient” rating for reliability. Green & Glasgow´s tool [88], the external validity dimension of the U.S. Preventive Services Task Force (USPSTF) manual [73] and the RITES tool [47] had a “very low” certainty of evidence for “sufficient” reliability.

#### Measurement error

Measurement error was reported for three tools. Two studies on measurement error of Gartlehner´s tool [86] and Loyka´s external validity framework [75], had an “adequate” methodological quality. Two studies on measurement error of the external validity dimension of the Downs & Black-checklist [22] had an “inadequate” methodological quality. However, all three tools had a “very low” certainty of evidence for “indeterminate” measurement error. Reasons for downgrading were risk of bias, indirectness, and imprecision due to small sample sizes.

#### Criterion validity

Criterion validity was reported only for Gartlehner´s tool [86]. Although there was no gold standard available to assess the criterion validity of this tool, the authors used expert opinion as the reference standard. The study assessing this measurement property had an “adequate” methodological quality. The overall certainty of evidence was “very low” for “sufficient” criterion validity due to risk of bias, imprecision, and indirectness.

#### Construct validity (hypotheses testing)

Five studies [22, 90, 91, 97, 98] reported on the construct validity of four tools. Three studies had a “doubtful” [90, 91, 98], one had an “adequate” [22] and one had a “very good” [97] methodological quality. The overall certainty of evidence was “very low” for three tools (mainly due to serious risk of bias, imprecision and serious indirectness) [22, 88, 98] and “low” for one tool (due to imprecision and serious indirectness) [47]. The “Selection-Bias”-dimension of the EPHPP tool [98] had “very low” certainty of evidence for “sufficient” construct validity and the RITES tool [47] had “low” certainty of evidence for “sufficient” construct validity. Both, the Green & Glasgow´s tool [88] and the Downs & Black-checklist [22], had “very low” certainty of evidence for “insufficient” construct validity.

Structural validity and cross-cultural validity were not assessed in any of the included studies.

## Discussion

### Summary and interpretation of results

To our knowledge this is the first systematic review identifying and evaluating the measurement properties of tools to assess the external validity of RCTs. A total of 28 tools were included. Overall, for more than half (n = 17/28, 61%) of the included tools the measurement properties were not reported. Only five tools had at least one “sufficient” measurement property. Moreover, the development process was not described in 14/28 (50%) of the included tools. Reliability was assessed most frequently (including inter-rater and/or test-retest reliability). Only three of the included tools had “sufficient” reliability (“very low” certainty of evidence) [47, 73, 88]. Hypotheses testing was evaluated in four tools, with half of them having “sufficient” construct validity (“low” and “very low” certainty of evidence) [47, 98]. Measurement error was evaluated in three tools, all with an “indeterminate” quality rating (“low” and “very low” certainty of evidence) [22, 75, 86]. Criterion validity was evaluated for one tool, having “sufficient” with “very low” certainty of evidence [86]. The RITES tool [47] was the measurement tool with the strongest evidence for validity and reliability. Its content validity, based on international expert-consensus, was “sufficient” with “moderate” certainty of evidence, while reliability and construct validity were rated as “sufficient” with “very low” and “low” certainty of evidence, respectively.

Following the three criteria for the recommendation of a measurement tool, all included tools were categorized as ‘B’. Hence, further research will be required for the recommendation for or against any of the included tools [26]. Sufficient internal consistency may not be relevant for the assessment of external validity, as the measurement models might not be fully reflective. However, none of the authors/developers did specify the measurement model of their measurement tool.

Specification of the measurement model is considered a requirement of the appropriateness for the latent construct of interest during scale or tool development [102]. It could be argued that researchers automatically expect their tool to be a reflective measurement model. E.g., Downs and Black [22] assessed internal consistency without prior testing for unidimensionality or structural validity of the tool. Structural validity or unidimensionality is a prerequisite for internal consistency [26] and both measurement properties are only relevant for reflective measurement models [103, 104]. Misspecification as well as lack of specification of the measurement model can lead to potential limitations when developing and validating a scale or tool [102, 105]. Hence, the specification of measurement models should be considered in future research.

Content validity is the most important measurement property of health measurement instruments [27] and a lack of face validity is considered a strong argument for not using or to stop further evaluation of a measurement instrument [106]. Only the RITES tool [47] had evidence of “sufficient” content validity. Nevertheless, this tool does not directly measure the external validity of RCTs. The RITES tool [47] was developed to classify RCTs on an efficacy-effectiveness continuum. An RCT categorized as highly pragmatic or as having a “strong emphasis on effectiveness” [47] implies that the study design provides rather applicable results, but it does not automatically imply high external validity or generalizability of a trial´s characteristics to other specific contexts and settings [107]. Even a highly pragmatic/effectiveness study might have little applicability or generalizability to a specific research question of review authors. An individual assessment of external validity may still be needed by review authors in accordance with the research question and other contextual factors.

Another tool which might have some degree of content or face validity is the indirectness-dimension of the GRADE method [92]. This method is a widely used and accepted method in research synthesis in health science [108]. It has been evolved over the years based on work from the GRADE Working Group and on feedback from users worldwide [108]. Thus, it might be assumed that this method has a high degree of face validity, although it has not been systematically tested for content validity.

If all tools are categorized as ‘B’ in a review, the COSMIN guidelines suggests that the measurement instrument “with the best evidence for content validity could be the one to be provisionally recommended for use, until further evidence is provided” [34]. In accordance with this suggestions, the use of the RITES tool [47] as an provisionally solution might therefore be justified until more research on this topic is available. However, users should be aware of its limitations, as described above.

### Implication for future research

This study affirms and supplements what is already known from previous reviews [9, 12, 14–18]. The heterogeneity of characteristics of tools included in those reviews was also observed in the present review. Although Dyrvig et al. [16] did not assess the measurement properties of available tools, they reported a lack of empirical support of items included in measurement tools. The authors of previous reviews could not recommend a measurement tool. Although their conclusions were mainly based on descriptive analysis rather than the assessment of quality of the tools, the conclusion of the present systematic review is consistent with them.

One major challenge on this topic is the serious heterogeneity regarding the terminology, criteria and guidance to assess the external validity of RCTs. Development of new tools and/or further revision (and validation) of available tools may not be appropriate before consensus-based standards are developed. Generally, it may be argued whether these methods to assess the external validity in systematic reviews of interventions are suitable [9, 12]. The experimental/statistical methods presented in Table 1 may offer a more objective approach to evaluate the external validity of RCTs. However, they are not feasible to implement in the conduction of systematic reviews. Furthermore, they focus mainly on the characteristics and generalizability of the study populations, which is insufficient to assess the external validity of clinical trials [109], since they do not consider other relevant dimensions of external validity such as intervention settings or treatment variables etc. [4, 109].

The methodological possibilities in tool/scale development and validation regarding this topic have not been exploited, yet. More than 20 years ago, there was no consensus regarding the definition of quality of RCTs. In 1998, Verhagen et al. [110] performed a Delphi study to achieve consensus regarding the definition of quality of RCTs and to create a quality criteria list. Until now, these criteria list has been a guidance in tool development and their criteria are still being implemented in methodological quality or risk of bias assessment tools (e.g. the Cochrane Collaboration risk of bias tool 1 & 2.0, the Physiotherapy Evidence Database (PEDro) scale etc.). Consequently, it seems necessary to seek consensus in order to overcome the issues regarding the external validity of RCTs in a similar way. After reaching consensus, further development and validation is needed following standard guidelines for scale/tool development (e.g. de Vet et al. [106]; Streiner et al. [111]; DeVellis [112]). Since the assessment of external validity seems highly context-dependent [9, 12], this should be taken into account in future research. A conventional checklist approach seems inappropriate [9, 12, 109] and a more comprehensive but flexible approach might be necessary. The experimental/statistical methods (Table 1) may offer a reference standard for convergent validity testing of the dimension “patient population” in future research.

This review has highlighted the necessity for more research in this area. Published studies and evaluation tools are important sources of information and should inform the development of a new tool or approach.

#### Strengths and limitations

One strength of the present review is the two-phase search method. With this method we believe that the likelihood of missing relevant studies was addressed adequately. The forward citation tracking using Scopus is another strength of the present review. The quality of the included measurement tools was assessed with an adapted and comprehensive methodology (COSMIN). None of the previous reviews has attempted such an assessment.

There are some limitations of the present review. First, a search for grey literature was not performed. Second, we focused on RCTs only and did not include assessment tools for non-randomized or other observational study design. Third, due to heterogeneity in terminology, we might have missed some tools with our electronic literature search strategy. Furthermore, it was challenging to find studies on measurement properties of some included tools, that did not have a specific name or abbreviation (such as EVAT). We tried to address this potential limitation by performing a comprehensive reference screening and snowballing (including forward citation screening).

## Conclusions

Based on the results of this review, no available measurement tool can be fully recommended for the use in systematic reviews to assess the external validity of RCTs. Several steps are required to overcome the identified difficulties before a new tool is developed or available tools are further revised and validated.

## Supplementary Information

**Additional file 1.**

**Additional file 2.**

**Additional file 3.**

**Additional file 4.**

## Data Availability

All data generated or analyzed during this study are included in this published article (and its supplementary information files).

## Abbreviations

CASP: Critical Appraisal Skills Programme
CCBRG: Cochrane Collaboration Back Review Group
CCT: controlled clinical trial
COSMIN: COnsensus based Standards for the selection of health Measurement Instruments
EPHPP: Effective Public Health Practice Project
EVAT: External Validity Assessment Tool
FAME: Feasibility, Appropriateness, Meaningfulness and Effectiveness
GATE: Graphical Appraisal Tool for Epidemiological Studies
GAP: Generalizability, Applicability and Predictability
GRADE: Grading of Recommendations Assessment, Development and Evaluation
HTA: Health Technology Assessment
ICC: intraclass correlation
LEGEND: Let Evidence Guide Every New Decision
NICE: National Institute for Health and Care Excellence
PEDro: Physiotherapy Evidence Database
PRECIS: PRagmatic Explanatory Continuum Indicator Summary
RCT: randomized controlled trial
RITES: Rating of Included Trials on the Efficacy-Effectiveness Spectrum
TREND: Transparent Reporting of Evaluations with Nonrandomized Designs
USPSTF: U.S. Preventive Services Task Force
