# Supplementary material for: Identification of tools used to assess the external validity of randomized controlled trials in reviews: a systematic review of measurement properties
Source: BMC Med Res Methodol. 2022 Apr 6;22:100. doi: 10.1186/s12874-022-01561-5 (PMC8985274; doi:10.1186/s12874-022-01561-5)
Supplement: Supplementary file 1 — Additional file 1. [file 12874_2022_1561_MOESM1_ESM.docx]

**Identification of tools used to assess the external validity of randomized controlled trials in reviews: A systematic review of measurement properties**

Andres Jung, Julia Balzer, Tobias Braun & Kerstin Luedtke

**Table S1: Search strategy for all databases**

**Search strings for Medline (via Pubmed)**:

Phase 1 search (March 2021):

| (((transferab*[tiab] OR “external valid*”[tiab] OR applicab*[tiab] OR indirectness[tiab] OR “model valid*”[tiab] OR representativeness[tiab] OR generalis*[tiab] OR generaliz*[tiab] OR extrapolation*[tiab])) AND (“randomized controlled trial*“[MH] OR RCT[tiab] OR RCTs[tiab])) AND (scale[tiab] OR scales[tiab] OR “critical appraisal“[tiab] OR “quality assessment“[tiab] OR tool[tiab] OR tools[tiab] OR instrument*[tiab] OR measure[tiab] OR measures[tiab] OR measurement*[tiab] OR framework*[tiab] OR questionnaire*[tiab] OR score[tiab] OR scores[tiab] OR checklist*[tiab] OR survey[tiab] OR surveys[tiab] OR rating*[tiab] OR index[tiab] OR indices[tiab] OR report[tiab] OR reports[tiab] OR reporting*[tiab]) Filters: Humans |
| --- |

Phase 2 search (March 2021):

| ((((Bornhoeft OR Bornhöft) OR ((CASP[tiab] OR "critical appraisal skills programme"[tiab]) AND ("Randomised Controlled Trial*"[tiab] OR RCT[tiab] OR RCTs[tiab])) OR ((GRADE[tiab] OR "Grading of Recommendations, Assessment, Development and Evaluation"[tiab]) AND (checklist[tiab] OR SAQAT[tiab] OR Meader)) OR Clegg OR ("Downs & Black" OR "Downs and Black" OR (Downs AND Black)) OR ("Effective Public Health Practice Project" OR EPHPP) OR EVAT OR (( JBI OR "Joanna Briggs Institute") AND (FAME OR (feasibility AND appropriateness AND meaningfulness AND effectiveness))) OR (("Grading of Recommendations, Assessment, Development and Evaluation"[tiab] OR GRADE[tiab]) AND indirectness[tiab]) OR ("Green & Glasgow" OR (Green AND Glasgow)) OR Haraldsson OR Karjalainen OR LEGEND[tiab] OR ("National Health and Medical Research Council"[tiab] OR NHMRC[tiab]) OR Sorg OR ("Van Tulder" OR "Cochrane Collaboration Back Review Group" OR CCBRG) OR ("Whole Systems Research") OR Liberati OR ("U.S. Preventive Services Task Force" OR USPSTF) OR (("The National Institute for Health and Care Excellence" OR NICE) AND GATE) OR (Cho AND bero) OR Loyka OR ((Carr OR (GAP AND predictability) OR Foy OR Estrada) AND ("randomized controlled trial*"[MH] OR RCT[tiab] OR RCTs[tiab]) AND (scale[tiab] OR scales[tiab] OR "critical appraisal"[tiab] OR "quality assessment"[tiab] OR tool[tiab] OR tools[tiab] OR instrument*[tiab] OR measure[tiab] OR measures[tiab] OR measurement*[tiab] OR framework*[tiab] OR questionnaire*[tiab] OR score[tiab] OR scores[tiab] OR checklist*[tiab] OR survey[tiab] OR surveys[tiab] OR rating*[tiab] OR index[tiab] OR indices[tiab] OR report[tiab] OR reports[tiab] OR reporting*[tiab]))) AND (transferab*[tiab] OR "external valid*"[tiab] OR applicab*[tiab] OR indirectness[tiab] OR “model valid*”[tiab] OR representativeness[tiab] OR generalis*[tiab] OR generaliz*[tiab] OR extrapolation*[tiab])) OR ((Gartlehner OR RITES) AND efficacy AND effectiveness)) |
| --- |
| AND |
| (instrumentation[sh] OR methods[sh] OR "Validation Study"[pt] OR "Comparative Study"[pt] OR "psychometrics"[MeSH] OR psychometr*[tiab] OR clinimetr*[tw] OR clinometr*[tw] OR "outcome assessment (health care)"[MeSH] OR "outcome assessment"[tiab] OR "outcome measure*"[tw] OR "observer variation"[MeSH] OR "observer variation"[tiab] OR "Health Status Indicators"[Mesh] OR "reproducibility of results"[MeSH] OR reproducib*[tiab] OR "discriminant analysis"[MeSH] OR reliab*[tiab] OR unreliab*[tiab] OR valid*[tiab] OR "coefficient of variation"[tiab] OR coefficient[tiab] OR homogeneity[tiab] OR homogeneous[tiab] OR "internal consistency"[tiab] OR (cronbach*[tiab] AND (alpha[tiab] OR alphas[tiab])) OR (item[tiab] AND (correlation*[tiab] OR selection*[tiab] OR reduction*[tiab])) OR agreement[tw] OR precision[tw] OR imprecision[tw] OR "precise values"[tw] OR test-retest[tiab] OR (test[tiab] AND retest[tiab]) OR (reliab*[tiab] AND (test[tiab] OR retest[tiab])) OR stability[tiab] OR interrater[tiab] OR inter-rater[tiab] OR intrarater[tiab] OR intra-rater[tiab] OR intertester[tiab] OR inter-tester[tiab] OR intratester[tiab] OR intra-tester[tiab] OR interobserver[tiab] OR inter-observer[tiab] OR intraobserver[tiab] OR intra-observer[tiab] OR intertechnician[tiab] OR inter-technician[tiab] OR intratechnician[tiab] OR intra-technician[tiab] OR interexaminer[tiab] OR inter-examiner[tiab] OR intraexaminer[tiab] OR intra-examiner[tiab] OR interassay[tiab] OR inter-assay[tiab] OR intraassay[tiab] OR intra-assay[tiab] OR interindividual[tiab] OR inter-individual[tiab] OR intraindividual[tiab] OR intra-individual[tiab] OR interparticipant[tiab] OR inter-participant[tiab] OR intraparticipant[tiab] OR intra-participant[tiab] OR kappa[tiab] OR kappa's[tiab] OR kappas[tiab] OR repeatab*[tw] OR ((replicab*[tw] OR repeated[tw]) AND (measure[tw] OR measures[tw] OR findings[tw] OR result[tw] OR results[tw] OR test[tw] OR tests[tw])) OR generaliza*[tiab] OR generalisa*[tiab] OR concordance[tiab] OR (intraclass[tiab] AND correlation*[tiab]) OR discriminative[tiab] OR "known group"[tiab] OR "factor analysis"[tiab] OR "factor analyses"[tiab] OR "factor structure"[tiab] OR "factor structures"[tiab] OR dimension*[tiab] OR subscale*[tiab] OR (multitrait[tiab] AND scaling[tiab] AND (analysis[tiab] OR analyses[tiab])) OR "item discriminant"[tiab] OR "interscale correlation*"[tiab] OR error[tiab] OR errors[tiab] OR "individual variability"[tiab] OR "interval variability"[tiab] OR "rate variability"[tiab] OR (variability[tiab] AND (analysis[tiab] OR values[tiab])) OR (uncertainty[tiab] AND (measurement[tiab] OR measuring[tiab])) OR "standard error of measurement"[tiab] OR sensitiv*[tiab] OR responsive*[tiab] OR (limit[tiab] AND detection[tiab]) OR "minimal detectable concentration"[tiab] OR interpretab*[tiab] OR ((minimal[tiab] OR minimally[tiab] OR clinical[tiab] OR clinically[tiab]) AND (important[tiab] OR significant[tiab] OR detectable[tiab]) AND (change[tiab] OR difference[tiab])) OR (small*[tiab] AND (real[tiab] OR detectable[tiab]) AND (change[tiab] OR difference[tiab])) OR "meaningful change"[tiab] OR "ceiling effect"[tiab] OR "floor effect"[tiab] OR "Item response model"[tiab] OR IRT[tiab] OR Rasch[tiab] OR "Differential item functioning"[tiab] OR DIF[tiab] OR "computer adaptive testing"[tiab] OR "item bank"[tiab] OR "cross-cultural equivalence"[tiab]) |

**Search strings for CINAHL via Ebsco:**

Phase 1 search (March 2021):

| TI ("external valid*" OR applicab* OR transferab* OR indirectness OR "model validity" OR representativeness OR generalisab* OR generalizab* OR generalize OR generalizing OR generalization OR generalisation OR generalise OR generalising OR extrapolation*) OR AB ("external valid*" OR applicab* OR transferab* OR indirectness OR "model validity" OR representativeness OR generalisab* OR generalizab* OR generalize OR generalizing OR generalization OR generalisation OR generalise OR generalising OR extrapolation*) |
| --- |
| AND |
| TI ("randomized controlled trial*" OR RCT*) OR AB ("randomized controlled trial*" OR RCT*) |
| AND |
| TI (scale OR scales OR "critical appraisal" OR "quality assessment" OR tool OR tools OR instrument* OR measure OR measures OR measurement* OR framework* OR questionnaire* OR score OR scores OR checklist* OR survey OR surveys OR rating* OR index OR indices OR report OR reports OR reporting*) OR AB (scale OR scales OR "critical appraisal" OR "quality assessment" OR tool OR tools OR instrument* OR measure OR measures OR measurement* OR framework* OR questionnaire* OR score OR scores OR checklist* OR survey OR surveys OR rating* OR index OR indices OR report OR reports OR reporting*) |

Phase 2 search (March 2021):

| ((TX Liberati OR TX "Whole Systems Research" OR TX Sorg OR TX "Let Evidence Guide Every New Decision" OR TX LEGEND OR TX Haraldsson OR TX "Green & Glasgow" OR TX "Green and Glasgow" OR (TX Green AND TX Glasgow) OR (TX "Fernandez-Hermida" AND TX GAP) OR TX Foy OR TX FAME OR TX (feasibility AND appropriateness AND meaningfulness AND effectiveness) OR TX EVAT OR TX Khorsan OR TX "Downs & Black" OR TX "Downs and Black" OR TX (Downs AND Black) OR TX Clegg OR TX Carr OR TX Bornhöft OR TX Bornhoeft OR TX "US Preventive Services Task Force" OR TX "U.S. Preventive Services Task Force" OR TX "United States Preventive Services Task Force" OR TX USPSTF OR TX Loyka OR TX Estrada) AND (TI ("external valid*" OR applicab* OR transferab* OR indirectness OR "model validity" OR representativeness OR generalisab* OR generalizab* OR generalize OR generalizing OR generalization OR generalisation OR generalise OR generalising OR extrapolation*) OR AB ("external valid*" OR applicab* OR transferab* OR indirectness OR "model validity" OR representativeness OR generalisab* OR generalizab* OR generalize OR generalizing OR generalization OR generalisation OR generalise OR generalising OR extrapolation*))) OR (TX NICE AND TX GATE) OR (TX "Effective Public Health Practice Project" OR TX EPHPP) OR ((TX "Grading of Recommendations, Assessment, Development and Evaluation" OR TX GRADE) AND TX indirectness) OR (TX Karjalainen AND TX "clinical relevance") OR ((TX "Van Tulder" OR TX "Cochrane Collaboration Back Review Group" OR TX CCBRG) AND TX "clinical relevance") OR ((TX "critical appraisal skills programme" OR TX CASP) AND (TX RCT* OR TX "randomized controlled trial*")) OR TX (Cho AND Bero) OR ((TX SAQAT OR TX checklist OR TX Meader) AND (TX GRADE OR TX "Grading of Recommendations, Assessment, Development and Evaluation") AND TX indirectness) OR (TX "National Health and Medical Research Council" OR TX nhmrc) AND (TX "external validity" OR TX applicability OR TX generalizability OR TX generalisability) OR ((TX Gartlehner OR TX RITES OR TX Wieland) AND TX (efficacy AND effectiveness)) |
| --- |
| AND |
| (MH “Psychometrics”) or ( TI psychometr* or AB psychometr* ) or ( TI clinimetr* or AB  clinimetr* ) or ( TI clinometr* OR AB clinometr* ) or (MH “Outcome Assessment”) or ( TI  outcome assessment or AB outcome assessment ) or ( TI outcome measure* or AB outcome  measure* ) or (MH “Health Status Indicators”) or (MH “Reproducibility of Results”) or (MH  “Discriminant Analysis”) or ( ( TI reproducib* or AB reproducib* ) or ( TI reliab* or AB reliab* )  or ( TI unreliab* or AB unreliab* ) ) or ( ( TI valid* or AB valid* ) or ( TI coefficient or AB  coefficient ) or ( TI homogeneity or AB homogeneity ) ) or ( TI homogeneous or AB  homogeneous ) or ( TI “coefficient of variation” or AB “coefficient of variation” ) or ( TI “internal  consistency” or AB “internal consistency” ) or (MH “Internal Consistency+”) or (MH  “Reliability+”) or (MH “Measurement Error+”) or (MH “Content Validity+”) or “hypothesis  testing” or “structural validity” or “cross-cultural validity” or (MH “Criterion-Related Validity+”) or “responsiveness” or “interpretability” or ( TI reliab* or AB reliab* ) and ( (TI test or AB test) OR  (TI retest or AB retest) ) or ( TI stability or AB stability ) or ( TI interrater or AB interrater ) or ( TI  inter-rater or AB inter-rater ) or ( TI intrarater or AB intrarater ) or ( TI intra-rater or AB intrarater  ) or ( TI intertester or AB intertester) or (TI inter-tester or AB inter-tester) or ( TI intratester  or AB intratester) or ( TI intra-tester or AB intra-tester) or ( TI interobserver or AB  interobserver) or (TI inter-observer or AB inter-observer ) or ( TI intraobserver or AB  intraobserver) or ( TI intra-observer or AB intra-observer) or ( TI intertechnician or AB  intertechnician) or (TI inter-technician or AB inter-technician) or ( TI intratechnician or AB  intratechnician ) or ( TI intra-technician or AB intra-technician ) or ( TI interexaminer or AB  interexaminer ) or (TI inter-examiner or AB inter-examiner) or (TI intraexaminer or AB  intraexaminer ) OR (TI intra-examiner or AB intra-examiner ) or (TI intra-examiner or AB intraexaminer) or (TI interassay or AB interassay ) or ( TI inter-assay or AB inter-assay ) or ( TI  intraassay or AB intraassay) or ( TI intra-assay or AB intra-assay ) or (TI interindividual or AB  interindividual) or (TI inter-individual or AB inter-individual) OR (TI intraindividual or AB  intraindividual) or (TI intra-individual or AB intra-individual) or (TI interparticipant or AB  interparticipant) or (TI inter-participant or AB inter-participant ) or (TI intraparticipant or AB  intraparticipant) or (TI intra-participant or AB intra-participant ) or (TI kappa or AB kappa) or (TI  kappa’s or AB kappa’s ) or (TI kappas or AB kappas) or (TI repeatab* or AB repeatab*) or ( TI  responsive* or AB responsive* ) or ( TI interpretab* or AB interpretab* ) |

**Search strings for PsychInfo via OVID:**

Phase 1 search (March 2021):

| (("external valid*" or applicab* or transferab* or indirectness or "model validity" or representativeness or generalisab* or generalizab* or generalize or generalizing or generalization or generalisation or generalise or generalising or extrapolation*) and ("randomized controlled trial*" or RCT*) and (Scale or scales or "critical appraisal" or "quality assessment" or tool or tools or instrument* or measure or measures or measurement* or framework* or questionnaire* or score or scores or checklist* or survey or surveys or rating* or index or indices or report or reports or reporting*)).ti,ab. |
| --- |

Phase 2 search (March 2021):

| (Liberati.ct. and "1986".cb. and ("quality" and ("randomized controlled trial*" or RCT or RCTs)).ti,ab.) or (Wieland.ct. and rites.ti,ab.) or "Whole systems research".ti,ab. or (Gartlehner.ct. and (tool or instrument or checklist or criteria or (efficacy and effectiveness)).ti,ab. and ("randomized controlled trial*" or rct or rcts).ti,ab.) or (sorg.ct. and ("external validity" or generalizability or generalisability or applicability).ti,ab.) or (("Let Evidence Guide Every New Decision" or legend).ti,ab. and Clark.ct.) or (karjalainen.ct. and "clinical relevance".ti,ab.) or (("Van Tulder".ct. or ("Cochrane Collaboration Back Review Group" or ccbrg).tw.) and "clinical relevance".ti,ab.) or (gate.tw. and ("The National Institute for Health and Care Excellence" or nice).ti,ab.) or (haraldsson.ct. and (clinical and applicability).ti,ab.) or ((green and Glasgow).ct. and ("external validity" or generalizability or generalisability or applicability).ti,ab.) or (("Grading of Recommendations, Assessment, Development and Evaluation" or grade) and indirectness).ti,ab,md. or (("Fernandez-Hermida" or "fernandez hermida").ct. and ("generalizability, applicability and predictability" or gap or "external validity").ti,ab.) or (Foy.ct. and "external validity".ti,ab.) or ((Pearson.ct. or (JBI or "Joanna Briggs Institute").tw.) and (FAME or "Feasibility, appropriateness, meaningfulness and effectiveness" or (feasibility and appropriateness and meaningfulness and effectiveness)).ti,ab.) or ((EVAT or "external validity assessment tool").ti,ab. or Khorsan.ct.) or (EPHPP or "Effective Public Health Practice Project").ti,ab. or (("Downs and Black" or "Downs & Black" or (Downs and Black)) and (checklist or tool or scale)).ti,ab. or (clegg.ct. and "external validity".ti,ab.) or (("critical appraisal skills programme" or casp) and ("Randomised Controlled Trial*" or rct or rcts)).ti,ab. or (carr.ct. and (applicability or generalizability or generalisability or "external validity").ti,ab. and (grade or (evidence and grading)).ti,ab.) or (bornhoeft.ct. and ("external validity" or "model validity").ti,ab.) or ((Cho and bero).ct. and ("clinical relevance" or instrument*).ti,ab.) or (((saqat or checklist) and (grade or "Grading of Recommendations, Assessment, Development and Evaluation")).ti,ab. and meader.ct.) or (("U.S. Preventive Services Task Force" or "US Preventive Services Task Force" or uspstf) and ("external validity" or applicability)).ti,ab. or (("National Health and Medical Research Council" or nhmrc) and ("external validity" or applicability or generalizability or generalisability)).ti,ab. or (Estrada.ct. and (applicability or transferability).tw.) or (Loyka.ct. and ("external validity" and framework).tw.) |
| --- |
| AND |
| *Translation of Pubmed search filter for PsychInfo via OVID, we refer to “Appendix. Search terms“ in:*  van der Hout A, Neijenhuijs KI, Jansen F, et al (2019) Measuring health-related quality of life in colorectal cancer patients: systematic review of measurement properties of the EORTC QLQ-CR29. Support care cancer Off J Multinatl Assoc Support Care Cancer 27:2395–2412; doi:10.1007/s00520-019-04764-7. |

**Search strings for Scopus:**

Phase 1 search (March 2021):

| ( TITLE-ABS ( "external valid*" OR applicab* OR transferab* OR indirectness OR "model validity" OR representativeness OR generalisab* OR generalizab* OR generalize OR generalizing OR generalization OR generalisation OR generalise OR generalising OR extrapolation* ) ) AND ( TITLE-ABS ( "randomized controlled trial*" OR rct* ) ) AND ( TITLE-ABS ( scale OR scales OR "critical appraisal" OR "quality assessment" OR tool OR tools OR instrument* OR measure OR measures OR measurement* OR framework* OR questionnaire* OR score OR scores OR checklist* OR survey OR surveys OR rating* OR index OR indices OR report OR reports OR reporting* ) ) AND ( EXCLUDE ( SUBJAREA , "SOCI" ) OR EXCLUDE ( SUBJAREA , "AGRI" ) OR EXCLUDE ( SUBJAREA , "ARTS" ) OR EXCLUDE ( SUBJAREA , "MATH" ) OR EXCLUDE ( SUBJAREA , "ENGI" ) OR EXCLUDE ( SUBJAREA , "COMP" ) OR EXCLUDE ( SUBJAREA , "ENVI" ) OR EXCLUDE ( SUBJAREA , "ECON" ) OR EXCLUDE ( SUBJAREA , "BUSI" ) OR EXCLUDE ( SUBJAREA , "CHEM" ) OR EXCLUDE ( SUBJAREA , "MATE" ) OR EXCLUDE ( SUBJAREA , "VETE" ) OR EXCLUDE ( SUBJAREA , "PHYS" ) OR EXCLUDE ( SUBJAREA , "CENG" ) OR EXCLUDE ( SUBJAREA , "EART" ) OR EXCLUDE ( SUBJAREA , "ENER" ) ) AND ( LIMIT-TO ( LANGUAGE , "English" ) OR LIMIT-TO ( LANGUAGE , "German" ) ) AND ( LIMIT-TO ( EXACTKEYWORD , "Human" ) OR LIMIT-TO ( EXACTKEYWORD , "Humans" ) ) |
| --- |

Phase 2 search (March 2021)

| ( ( REF ( liberati 1986 ) AND TITLE-ABS-KEY ( quality ) AND TITLE-ABS-KEY ( evaluation OR assessment ) AND TITLE-ABS-KEY ( "randomized controlled trial*" OR rct OR rcts ) ) OR ( TITLE-ABS-KEY ( "Rating of Included Trials on the Efficacy-effectiveness Spectrum" OR rites ) AND REFAUTH ( wieland ) ) OR ( TITLE-ABS-KEY ( "Whole systems research" )) OR ( REF ( gartlehner 2006 ) AND TITLE-ABS-KEY ( tool OR instrument OR checklist OR criteria ) AND TITLE-ABS-KEY ( "randomized controlled trial*" OR rct OR rcts ) ) OR ( REF ( sorg 2009 ) AND TITLE-ABS-KEY ( "external validity" OR generalizability OR generalisability OR applicability ) ) OR ( REF ( clark 2009 ) AND TITLE-ABS-KEY ( "Let Evidence Guide Every New Decision" OR legend ) ) OR ( REF ( karjalainen 2000 ) AND TITLE-ABS-KEY ( "clinical relevance" ) ) OR ( REF ( "Van Tulder" 2003 ) OR ALL ( "Cochrane Collaboration Back Review Group" OR ccbrg ) AND TITLE-ABS-KEY ( "clinical relevance" ) ) OR ( TITLE-ABS-KEY ( "Graphic Appraisal Tool for Epidemiological studies" OR gate ) AND TITLE-ABS-KEY ( "The National Institute for Health and Care Excellence" OR nice ) ) OR ( REF ( haraldsson 2006 ) AND TITLE-ABS-KEY ( clinical AND applicability ) ) OR ( REF ( green 2006 ) AND REF ( glasgow 2006 ) AND TITLE-ABS-KEY ( "external validity" OR generalizability OR generalisability OR applicability ) AND TITLE-ABS-KEY ( rating OR criteria ) ) OR ( TITLE-ABS-KEY ( "Grading of Recommendations, Assessment, Development and Evaluation" OR grade ) AND TITLE-ABS-KEY ( indirectness ) ) OR ( REF ( "Fernandez-Hermida" 2012 ) AND TITLE-ABS-KEY ( "generalizability, applicability and predictability" OR gap OR "external validity" ) ) OR ( REF ( foy 2010 ) AND TITLE-ABS-KEY ( "external validity" ) ) OR ( ( REFAUTH ( pearson ) OR ALL ( jbi OR "Joanna Briggs Institute" ) ) AND TITLE-ABS-KEY ( fame OR "Feasibility, appropriateness, meaningfulness and effectiveness" ) ) OR ( TITLE-ABS-KEY ( evat OR "EV assessment tool" OR "external validity assessment tool" ) ) OR ( TITLE-ABS-KEY ( ephpp OR "Effective Public Health Practice Project" ) ) OR ( TITLE-ABS-KEY ( "Downs and Black" OR "Downs & Black" ) AND TITLE-ABS-KEY ( checklist OR tool OR scale ) ) OR ( REF ( clegg 2001 ) AND TITLE-ABS-KEY ( "external validity" ) ) OR ( TITLE-ABS-KEY ( "critical appraisal skills programme" OR casp ) AND TITLE-ABS-KEY ( "Randomised Controlled Trial*" OR rct OR rcts ) ) OR ( REF ( carr 2004 ) AND TITLE-ABS-KEY ( applicability OR generalizability OR "external validity" ) AND TITLE-ABS-KEY ( grade OR ( evidence AND grading ) ) ) OR ( REF ( bornhöft 2006 ) AND TITLE-ABS-KEY ( "external validity" OR "model validity" ) ) OR ( REF ( estrada 2021 ) AND TITLE-ABS-KEY ( applicability OR transferability ) ) OR ( REF ( loyka 2020 ) AND TITLE-ABS-KEY ( framework ) ) OR ( REF ( cho 1994 ) AND REF ( bero 1994 ) AND TITLE-ABS-KEY ( "clinical relevance" ) ) OR ( TITLE-ABS-KEY ( ( saqat OR "semi-automated quality assessment tool" OR checklist ) AND ( grade OR "Grading of Recommendations, Assessment, Development and Evaluation" ) ) AND REF ( meader ) ) OR ( TITLE-ABS-KEY ( ( "U.S. Preventive Services Task Force" OR "US Preventive Services Task Force" OR uspstf ) AND ( "external validity" OR applicability ) ) ) OR ( TITLE-ABS-KEY ( ( "National Health and Medical Research Council" OR nhmrc ) AND ( "external validity" OR applicability ) ) ) ) |
| --- |
| AND |
| *Translation of Pubmed search filter for Scopus, we refer to “Supplementary material 1“ in:*  Sierevelt IN, Zwiers R, Schats W, Haverkamp D, Terwee CB, Nolte PA, Kerkhoffs GMMJ (2018) Measurement properties of the most commonly used Foot- and Ankle-Specific Questionnaires: the FFI, FAOS and FAAM. A systematic review. Knee Surg Sports Traumatol Arthrosc 26:2059–2073; doi:10.1007/s00167-017-4748-7. |
