# Supplementary material for: Identification of tools used to assess the external validity of randomized controlled trials in reviews: a systematic review of measurement properties
Source: BMC Med Res Methodol. 2022 Apr 6;22:100. doi: 10.1186/s12874-022-01561-5 (PMC8985274; doi:10.1186/s12874-022-01561-5)
Supplement: Supplementary file 2 — Additional file 2. [file 12874_2022_1561_MOESM2_ESM.docx]

**Identification of tools used to assess the external validity of randomized controlled trials in reviews: A systematic review of measurement properties**

Andres Jung, Julia Balzer, Tobias Braun & Kerstin Luedtke

| **Table S2: Specified COSMIN rating rules for the measurement properties of tools to assess the external validity of randomized controlled trials** | |
| --- | --- |
| **Item** | **specified COSMIN rule** |
| For all Boxes | |
| Item ‘Were there any important flaws in the design or methods of the study?’ | - if unexplained missing data is detected |
| For Boxes 2 – 9: only data of the final version of the measurement tool is being assessed. Data from pilot versions should be considered part of the development process (Box 1).  - Decision for “final version”:  If it is unclear whether data was derived from a final version, the reviewers must decide, depending on the reporting of the article, whether it can be assumed that the data was derived from the final version or if no further modifications/adaptations were made after pilot testing.  - For all Boxes: A PROM, in our context, is a reviewer-reported measurement tool | |
| **Box 1: Measurement tool development**  **Box 1a + 1b need to be evaluated only once per tool (development study of the original tool). Modified tools will be treated as new tools and have to be evaluated in Box 1 + 2. If pilot tests were performed in context of a cross-cultural adaptation, this will be evaluated in Box 5 (not Box 1b), as it is an essential component of the cross-cultural adaptation according to Beaton et al. (2000).** | |
| Item 01 “Is a clear description provided of the  construct to be measured?  &  Item 02 “Is the origin of the construct clear: was a theory, conceptual framework or disease model used or clear rationale provided to define the construct to be measured? | - if description is poor, at least a reference to the literature must be available to specify the construct of interest, otherwise this item will be rated as “inadequate” |
| Item 03 “Is a clear description provided of the target population for which the PROM was developed?” | - report quality only (rated such as Item 1&2) |
| Item 04 “Is a clear description provided of the context of use?” | - report quality only (rated such as Item 1&2) |
| **Item 05** “Was the PROM development study performed in a sample representing the target population for which the PROM was developed?” | - Target population(s) in our context are two:  1. Target study design: RCTs  2. Target population: authors of systematic reviews of interventions  - if this item 05 is scored as *“inadequate”: tool development is inadequate, no further rating required for Box 1a (in accordance with the COSMIN manual)* |
| Item 06 “Was an appropriate qualitative data collection method used to identify relevant items for a new PROM?” | - appropriate data collection methods for item-generation process according to De Vet et al. (2011; p. 37) are inductive and deductive. In our context:  Inductive: Data from expert-panels, interviews with clinicians/experts/researchers, focus groups. If such an approach was applied a moderator is needed. In addition, we consider written feedback from clinicians, or researchers/experts (e.g. Delphi study) for the inclusion of possible relevant items as appropriate (no moderator is needed, items 7-9 can be skipped).  Deductive (literature): scoping reviews, systematic reviews (to identify existing tools) and data/evidence from meta-epidemiology (if available)  Note: qualitative data collection from patients is a necessary (inductive) method for item generation within the development process of a patient reported outcome measurement tool. Since this is not possible for our construct/target population of interest (RCTs, no patients), we therefore consider the following evaluation criteria:  If both methods (inductive and deductive) were applied and well described, we will give a “very good” rating. If only deductive methods were applied and well described, we will give an “adequate” rating and items 07-13 will be skipped. |
| Item 07 “Were skilled group moderators/ interviewers used?” | - skilled interview and pilot testing of the interview guide: item 07 is scored as *“very good”*  *-* if it is not clear if a trained moderator performed the interview: item 07 is scored as *“doubtful”*  *-* if only qualitative written feedback was provided: item 07-09 will be scored as *“Not applicable” and scoring will be continued with item 10-12 in terms of the qualitative written feedback* |
| Item 10 “Was an appropriate approach used to analyse the data?” | - appropriate data (*qualitative)* analysis only relevant for tool development/item generation (not reliability/validity) examples of qualitative methods are listed in the COSMIN manual for assessing methodology of content validity of PROMS (p.24) |
| Item 11” Was at least part of the data coded independently?” | - only applicable if qualitative data was obtained, e.g. with open ended questions (interview or written feedback). Otherwise “N/A” |
| Item 12 “Was data collection continued until saturation was reached?” | - appropriate method for data saturation needs to be explicitly described: saturation table, matrix, grid: *item 12 is scored as “very good”*  *- if data saturation was only suggested, item 12 is scored as “adequate”* |
| Item 13 “For quantitative studies (surveys): was the sample size appropriate?” | - sample size of the quantitative survey is rated according to the COMSIN manual  ≥100 “*very good”*  *50-99 “adequate”*  *30-49 “doubtful”*  *˂30 “inadequate”* |
| **Pilot-test** | |
| Item 14 “Was a cognitive interview study or other pilot test conducted?” | - this item only evaluates whether a pilot test was performed – and not how well this was described/performed,  - if **yes**, *item 14 is scored as “very good”*  - if **not,** *item 14 is scored as “inadequate”* and the rest of the box can be skipped and the total quality of the non-PROM development study will be rated as *“inadequate”* |
| Item 15 “Was the cognitive interview study or other pilot test performed in a sample representing the target population?” | - Target population(s) in our context are two:  1. Target study design: RCTs  2. Target population: authors of systematic reviews of intervention studies  - piloting should be performed on RCTs  - if this was not clearly described in the study, *item 16 is scored as “doubtful” and items 16-25 can be skipped.* |
| Item 16 “Were patients asked about the  comprehensibility of the PROM?“ | - this item only evaluates whether clinicians or researchers/experts were asked about comprehensibility and not how well this was described/performed,  - if **yes**, *item 16 is scored as “very good”*  - if **not (or not clear),** *item 16 is scored as “inadequate”,* the boxes 17-25 can be skipped and the total quality of the PROM development study will be rated as *“****doubtful” or "inadequate”*** |
| Item 17 “Were all items tested in the final version” | If items were omitted or adapted after pilot-testing, the new version of the tool needs to be evaluated again,  - if this is assumable, item 17 is scored as *“adequate”,*  *-* if it is clearly described*, item 17 is scored “very good”* |
| Item 18 “Was an appropriate qualitative method used to assess the comprehensibility of the PROM instructions, items, response options, and recall period? | - appropriate qualitative data collection methods for comprehensibility are described in the COSMIN manual for assessing methodology of content validity of PROMS (p.29)  - this item only evaluates whether the chosen qualitative method was appropriate – it does not assess how well this method was applied in the study.  - for our research question, we consider written feedback about the comprehensibility of the tool from study participants (clinicians and/or researchers/experts) as “*adequate”* (in contrast to the manual), e.g. from Delphi study (items 20-22 can be skipped)  - if the study does not describe which kind of qualitative method was applied, item 18 is scored as “*doubtful”*  - verbal feedback/ interviews (etc.) with participants via telephone or face-to-face-meetings is scored “*very good”* |
| Item 26 “Were patients asked about the comprehensiveness of the PROM?*”* | - this item only evaluates whether participants (clinicians and/or researchers/experts) were asked about comprehensiveness of the measurement tool – and not how well this was described/performed,  - if **yes**, *item 26 is scored as “very good”*  - if **not or not clearly descibed,** *item 26 is scored as “doubtful”* and boxes 27-35 can be skipped |
| Item 28 “Was an appropriate method used for assessing the comprehensiveness of the PROM? | - appropriate qualitative data collection methods for comprehensiveness are described in the COSMIN manual for assessing methodology of content validity of PROMS (p.33/29)  - this item only evaluates whether the chosen qualitative method was appropriate – it does not assess how well this method was applied in the study.  - for our construct of interest, we consider written feedback about the comprehensibility of the tool from study participants (clinicians and/or researchers/experts) as “adequate” (in contrast to the manual), e.g. from Delphi study (items 30-32 can be skipped)  - if the study does not describe which kind of qualitative method was applied, item 28 is scored as “*doubtful”*  - verbal feedback/ interviews (etc.) with participants via telephone or face-to-face-meetings is scored “*very good”* |
| **Box 2:** **Content Validity**  - only assessed if the final version of the developed tool was tested on its content validity (it does *not* assess the development process (qualitative study and quantitative pilot testing) of the tool, content validity must be tested in a different sample (not the same sample as PROM development: Box 1)  Supplement “final version”: If content validation has been performed and after further evaluation/validation process some items have been deleted but none of the remaining items have been modified and content validation has not been performed again: only “comprehensiveness” will be rated as doubtful or inadequate (depending on the information available). If items have been deleted because of redundancy, we will consider to not downgrade “comprehensiveness”  If content validation has been performed and after further evaluation/validation process some items have been modified and content validation has not been performed again: “relevance”, “comprehensibility” and “comprehensiveness” will be rated as doubtful or inadequate (depending on the information available and/or degree of modification). If only minor modifications have been done (e.g. substitution of a word by synonym for better comprehensibility or rewording of items for a different target population), we will consider to not downgrade the content validity    - each of the 5 boxes (2a-e) can be rated separately – only rate the sub-boxes which were described in the study, overall rating of content validity should be determined per sub-box separately | |
| 2a-2c. “Asking patients about the relevance/comprehensiveness/comprehensibility of the PROM items  2d. “Asking professionals about the relevance of the PROM items”  2e. “Asking professionals about the comprehensiveness of the PROM” | Patients in our context are clinicians or researchers, who perform systematic reviews/meta-analysis and have to assess the quality of RCTs (e.g. user-panel).  Professionals in our context, are experts in the field of meta-research, trial methodology research, tool-development, epidemiology and meta-epidemiology  Comprehensiveness-assessment has to be described (“very good”) or it has to be assumed that comprehensiveness was assessed to achieve “adequate”. |
| Item 01 “Was an appropriate method used to ask patients whether each item is relevant for their experience with the condition?  Item 08 “Was an appropriate method used for assessing the comprehensiveness of the PROM?”  Item 15 “Was an appropriate qualitative method used for assessing the comprehensibility of the PROM instructions, items, response options, and recall period?”  Item 22 “Was an appropriate method used to ask professionals whether each item is relevant for the construct of interest?”  Item 27 “Was an appropriate method used for assessing the comprehensiveness of the PROM?” | - appropriate (qualitative) data collection are described in the COSMIN manual for assessing methodology of content validity of PROMS (p.37), the study should explicitly describe that relevance, comprehensibility and comprehensiveness were assessed for content validity.  - these items only evaluate whether the chosen qualitative/quantitative method was appropriate – it does not assess how well this method was applied in the study.  - for our research question, we consider written feedback about the relevance/comprehensiveness/comprehensibility of the tool´s items from study participants (clinicians or researchers/experts) as “adequate” (in contrast to the manual), e.g. Delphi study  verbal feedback/interviews (via face-to-face-meetings or via telephone), focus groups (etc.) with clinicians or researchers/experts will be scored as “very good”  - if the study does not describe which kind of qualitative/quantitative method was applied, “*doubtful” should be scored* |
| **Box 3. Structural validity** | |
| “Does the scale consist of effect indicators, i.e. is it based on a reflective model? yes / no” | *-* scoring of this item will be based on the author´s description.  Due to the still lacking consensus about adequate model formation (Jarvis, MacKenzie & Podsakoff, 2003; Hardin, Chang & Fuller, 2008; Marakas, Johnson & Clay, 2008), we will *not* evaluate if the chosen model is correct. This evaluation would need a more comprehensive (quantitative and qualitative) and complex approach. |
| **Box 4: Internal Consistency** | |
| Item 02. “For continuous scores: Was Cronbach’s alpha or omega calculated?” | - we consider, Cronbach´s Alpha appropriate for ordinal sum scores as well (Norman, 2010) |
| **Box 5:** **Cross-Cultural Validity**  **- COSMIN items evaluate the statistical quality of the cross-cultural validity**  **- in addition the guidelines in according to Beaton, 2000 should be rated under item 04** | |
| Item 02 “Was an appropriate approach used to analyse the data?” | - appropriate quantitative analysis method for cross-cultural validity of tools are: CFA, Regressions-Analysis, IRT  - if no quantitative analysis was performed, only translation according to Beaton et al. (2000) or other equivalent guideline for cross-cultural adaptation/translation, Item 02 will be rated as *“doubtful”* |
| Item 03 “Was the sample size included in the analysis adequate?” | - for quantitative approach: as described in the COSMIN manual  - for qualitative analysis in accordance with Beaton et al. (2000) a sample of 30-40 is adequate |
| Item 04 “Were there any other important flaws in the design or statistical methods of the study?” | - if translation process is NOT explicitly described in accordance to guidelines for cross cultural adaptation, e.g. guideline of Beaton et al. (2000), item 04 will be rated as “*inadequate”. A standardized process should be described that contains certain key elements of a cross-cultural adaptation, like forward-backward translation and pre-testing (pilot testing).*  - if cultural adaptations are NOT (clearly) described, item 04 will be rated as “*inadequate”* |
| **Box 6:** **Reliability** | |
| Item 01 “Were patients stable in the interim period on the construct to be measured?”  Item 02 “Was the time interval appropriate?”  Item 03 “Were the test conditions similar?’ e.g. type of administration, environment, instructions” | - target population: RCTs are stable, since they don´t change. Patients: Clinicians and/or researchers are expected to be stable: N/A    - The time interval for test-retest (or intra-rater reliability) testing should have taken place within the general recommended time interval of about 2 weeks (Mokkink et al., 2018; p. 54), in order to minimize bias in relation to a recall-bias.  - If only inter-rater reliability was tested, item 02 is going to be rated as: “Not applicable” (in contrast to the COSMIN manual)  - We do not expect any risk of bias for this item. Therefore, we won´t give an “inadequate” or “doubtful” rating if no information is stated on test conditions. We will consider to give an “inadequate” rating if e.g. ongoing adjustments of the tool have been made during the reliability testing |
| Item 04 “For continuous scores: Was an intraclass correlation coefficient (ICC) calculated?” | - In addition to the ICC we consider the Krippendorff´s alpha (Hayes & Krippendorff, 2007) as appropriate reliability statistics (De Swert, 2012; Hallgren, 2012) for continuous and/or ordinal sum-scores. To score “very good” the model or formula of the Krippendorff´s Alpha has to be described. If model or formula is not described: “adequate”  - Sum-Scores of ordinal items/domains may be treated as continuous scores and individual items are treated as ordinal or nominal/dichotomous scores. |
| Item 06 “For ordinal scores: Was a weighted kappa calculated?”  Item 07 “For ordinal scores: Was the weighting scheme described? e.g. linear, quadratic” | In accordance to Fleiss & Cohen (1973) and Norman (2010) robustness of the ICC when used with ordinal scaled data up to 7 response categories is high.  - if the ICC was applied for ordinal scales with 7 or more response categories, item 06 will be rated as *“very good*” and item 7 will be rated as “very good” if model or formula of the ICC is described, “adequate” if model or formula of the ICC is not described (as in item 04)  - if the ICC was applied for ordinal scales with less than 7 response categories, item 06 and 7 will be rated as *“inadequate”*  In accordance to De Swert (2012) and Hallgren (2012) we consider the ICC (response categories of ≥ 7) and the Krippendorff´s alpha (Hayes & Krippendorff, 2007) in addition to the weighted kappa, to be an appropriate reliability statistics method for ordinal scale (summed) scores as well. |
| Item 08 “Were there any other important flaws in the design or statistical methods of the study?” | - final version not tested for reliability (if items have been modified or deleted after reliability testing, reliability has to be tested again) 🡪 Box 1; if only very minor modifications were done, the reliability of the tool might be rated in this box. In this case, a “doubtful” rating will be considered. |
| **Box 7:** **Measurement Error**  **This box will be only rated for continuous scores the Standard Error of Measurement (SEM), Smallest Detectable Change (SDC) or Limits of Agreement (LoA) and for dichotomous/nominal/ordinal scores the percentage (positive and negative) agreement was calculated. Items 1,2 and 3 will be rated like items in Box 6** | |
| **Box 8. Criterion validity**  **Since there is no gold standard tool available for the assessment of external validity of RCTs, it is not expected that a study has used this approach to validate its tool. However, if authors used expert consensus methods (e.g. Delphi study) as a reference/gold standard, we will evaluate this box.** | |
| **Box 9:** **Hypotheses Testing**  **This box will be rated when convergent and discriminative validity were tested.** | |
| Item 01 “Is it clear what the comparator instrument(s) measure(s)?”  Item 05 “Was an adequate description provided of important characteristics of the subgroups?” | - If a comparator measurement tool or group was chosen to investigate construct validity, the comparator should be described in detail or references should be included |
| Item 02 “Were the measurement properties of the comparator instrument(s) sufficient?”  Item 04/07 “Were there any other important flaws” | - For measurement properties of comparator tools (convergent validity) described in detail (and sufficient): very good;  only references (and sufficient): adequate.  Considerations, why this tool/group has been chosen should be described in the study as well.  - If the article did not state an explicit hypothesis and its expected magnitude (i.e. only the aim/purpose of the study):   - item 04 should be rated as “adequate” or “doubtful” (for convergent validity a positive correlation with a measurement tool assessing similar constructs can be expected; this hypothesis may not need to be specified; in such case score: “adequate”) - Item 07 (discriminative validity) should be rated as *‘doubtful’* |
| **Hypotheses for review** | - Hypotheses for convergent validity: positive correlation (≥ 0.5, according to Prinsen et al., 2018) of the tool to be tested with another tool or approach (e.g. data from epidemiological studies) measuring a similar construct.  - Hypotheses for discriminative validity: correlations (≥ 0.5) or significant differences between reviewer-reported ratings of reviewers from different regions/countries. Correlations (≥ 0.5) or significant differences of ratings of an eligible tool between pragmatic RCTS and explanatory RCTs (classification of RCTs defined by an expert group or a tool (e.g. PRECIS tool)) or RCTs from different medical fields or settings (e.g. pharmacological versus non-pharmacological). |
| **Box 10:** **Responsiveness**  **N/A**  **- Responsiveness according to COSMIN is described as: “The ability of an HR-PRO instrument to detect change over time in the construct to be measured”.**  **For our construct of interest “external validity of RCTs”, responsiveness cannot be measured, since RCTs (and their validity) do not change over time. Therefore, Box 10 will not be evaluated and will be rated as “not applicable”.** | |
