# Supplementary material for: Identification of tools used to assess the external validity of randomized controlled trials in reviews: a systematic review of measurement properties
Source: BMC Med Res Methodol. 2022 Apr 6;22:100. doi: 10.1186/s12874-022-01561-5 (PMC8985274; doi:10.1186/s12874-022-01561-5)
Supplement: Supplementary file 3 — Additional file 3. [file 12874_2022_1561_MOESM3_ESM.docx]

**Identification of tools used to assess the external validity of randomized controlled trials in reviews: A systematic review of measurement properties**

Andres Jung, Julia Balzer, Tobias Braun & Kerstin Luedtke

**Table S 3: Excluded tools and reports**

| **Excluded tools (Phase 1)** | | |
| --- | --- | --- |
| **Name of tool** | **Authors** | **Reasons for exclusion** |
| Agency for Healthcare Research and Quality (AHRQ) criteria for applicability | Atkins et al. (2011) | Not considered a measurement tool. A list of relevant questions (taken from AHRQ 2007/2008, Chapter 6, p. 45) on factors affecting external validity is presented, but no methodology on assessment of external validity. |
| Appraisal of Guidelines for REsearch & Evaluation (AGREE) II, applicability-dimension | Brouwers et al. (2010, updated 2017) | Wrong target study design. Tool for assessment of quality of clinical guidelines with a domain for applicability. But assessment of individual RCTs not possible |
| Ahmad´s extraction tool | Ahmad et al. (2009) | Not a measurement tool to assess the external validity of RCTs. |
| Association of periOperative Registered Nurses (AORN) Research Evidence Appraisal Tool | Spruce et al. (2014) | Not a measurement tool to assess the external validity of RCTs. |
| Applicability Scoring of Surgical trials. An Investigator-reported aSsessment (ASSIST) Tool | Tourabaly et al. (2012) | Tool to assess the applicability of results of surgical RCTs from the trialist´s perspective. Not suitable for authors of systematic reviews and only applicable for surgical RCTs. |
| Augustovski´s assessment of generalizability of economic evaluation | Augustovski et al. (2009) | Does not measure construct of interest. Tool to assess generalizability of economic evaluation studies in Latin America. |
| Bizzini grading Scale | Bizzini et al. (2003) | Tool contains items concerning internal and external validity in each domain, but external validity cannot be assessed individually. |
| Bonell´s framework | Bonell et al., (2006) | Not considered a measurement tool. Method more focused on report quality of Generalizability factors for discussion from policymaker´s point of view |
| Brien´s Assessment | Brien et al., (2006) | No adequate description of tool. Developed specifically for patients with osteoarthritis. |
| Cartwright-Hatton´s criteria | Cartwright-Hatton et al. (2004) | Not a measurement tool to assess the external validity of RCTs. |
| Consensus on Health Economic Criteria (CHEC)-List | Evers et al., (2005) | Tool focuses on methodological quality of economic studies. Tool contains items of external validity, but an assessment of external validity cannot be done individually. |
| Consolidated Health Economic Evaluation  Reporting Standards (CHEERS) statement | Husereau et al. (2013) | Not a measurement tool to assess the external validity of RCTs. Tool to measure the report quality of health economic evaluation |
| Chen´s quality criteria | Chen et al., (2014) | Not a measurement tool to assess the external validity of RCTs. It is specifically adapted for psychotherapy, therefore not applicable in other medical fields. |
| Cheng´s quality assessments | Cheng, (2009) | Tool or method not clearly described. It is designed to measure the report quality. |
| Coleman Methodology Score | Coleman et al. (2000) | Although the tool contains items relevant to external validity, external validity cannot be assessed individually.  Tool is specifically designed for "Studies of surgical outcome after patellar tendinopathy". |
| Dekkers´ approach to assess external validity and applicability | Dekkers et al., 2009 | Not considered a measurement tool. No response options/categories presented.  Rather a “table with relevant aspects that should be considered, when assessing external validity and applicability” (Dekkers et al. 2009) |
| Dutch Cochrane Centre methodological quality criteria | cited by Vermeulen et al. (2004) | Tool is not clearly described. This tool is no longer available on the Dutch Cochrane website.  Tool seems to measure only methodological quality. |
| Effective Practice and Organization of Care (EPOC) | Cochrane Effective Practice and Organization of Care | Tool measures RoB (internal validity). |
| Graphical appraisal tool for epidemiological studies (GATE) | Jackson (2006) | Not a measurement tool to measure the external validity of RCTs. |
| Generalizability Index (Gix) | Gheorghe et al. (2015) | No tool or checklist. It is a statistical approach of quantitative measure of representativeness. |
| Goedhard´s generalizability criteria | Goedhard et al. (2006) | Only applicable for trials on pharmacotherapy for psychiatric patients. |
| Interventional Pain Management techniques Quality Appraisal of Reliability and Risk of Bias Assessment (IPM-QRB) | Manchikanti et al. (2014) | Not a measurement tool to measure the external validity of RCTs. Wrong study design (nonrandomized studies). Developed specifically for pain management. |
| Jennings´external validity assessment | Jennings et al. (2014) | External validity criteria were included in the quality assessment. However, no further information on items/questions, rating criteria, response options/categories and development was available in this review article. The corresponding author was contacted for more information but did not respond. |
| Jonsson´s checklist | Jonsson et al. (2016) | Not considered a measurement tool. Method focused on report quality of factors relevant to external validity. |
| Kahan´s criteria for generalizability scores | Kahan et al. (1995) | Tool only applicable for trials of physician-based interventions with problem drinkers. |
| Kienle´s Kriterien für Praxisrelevanz/Modellvalidität | Kienle et al. (2006) | Tool only applicable for anthroposophic medicine |
| McMaster Critical review Form-quantitative studies | Law et al. (1998) | Not a measurement tool to assess the external validity of RCTs. Tool to measure the methodological quality of quantitative studies. It does not measure external validity. |
| modified AHRQ method; external validity-dimension | Hartmann et al. (2009) | Tool focused only on report quality. It contains items for internal validity |
| Methodical Quality Index (MQI) | Dantas et al. (2007) | Not a measurement tool to assess the external validity of RCTs. Tool aims to assess methodological quality. |
| Model Validity of Homeopathic Treatment (MVHT) | Mathie et al. (2012) | Tool only applicable for homeopathic treatments. |
| Perlow´s scoring scheme of methodological quality | Perlow et al. (2002) | Tool aims to assess methodological quality. It contains items relevant to both, internal and external validity, but external validity cannot be assessed individually. |
| Psychotherapy outcome study methodological rating form (POMRF) | Öst (2007) | The tool contains items relevant to report quality, internal validity and external validity, but external validity cannot be assessed individually. |
| PRagmatic Explanatory Continuum Indicator Summary (PRECIS) 2 | Loudon et al. (2015) | Tool developed for the planning stage of an RCT (**prospective**). “…tool to help trialists make design decisions consistent with the intended purpose of their trial…” (Loudon et al., 2015). It is not designed to assess the external validity of an RCT retrospectively and not suitable for authors of systematic reviews. (This is one of the reasons, why the RITES tool was developed.) |
| Purepong´s external validity checklist | Purepong et al. (2012) | Tool only applicable for acupuncture research. |
| RCT-Psychotherapy Quality Rating Scale (RCT-PQRS) | Kocsis et al. (2010) | Not a measurement tool to assess the external validity of RCTs. Tool aims to assess methodological quality (report quality + internal validity). |
| Reach, Effectiveness,  Adoption, Implementation, and Maintenance (RE-AIM) framework | Glasgow et al. (1999) | Tool developed for implementation research. It was developed for planning and reporting on key issues related to implementation and external validity. It is not designed for retrospective assessment of RCTs. This is one of the reasons why the authors later presented a list of criteria to assess reporting of external validity of clinical trials based on the Re-Aim framework (Green & Glasgow, 2006). |
| Rothwell´s list of issues potentially affecting external validity | Rothwell (2005) | Not considered a measurement tool. A list of potential issues regarding external validity of RCTs is presented. |
| Scottish Intercollegiate Guidelines Network (SIGN) 50 manual | SIGN50 (2001) manual | No measurement tool to assess external validity provided. The manual encourages authors of SIGN guidelines to comment on generalizability. |
| Soares´ critical appraisal of applicability of results | Soares et al. (2002) | Not considered a measurement tool. Only list of relevant questions to external validity is provided. |
| Tahmosybayat´s adapted quality assessment tool | Tahmosbayat et al. (2017) | Not a measurement tool to assess the external validity of RCTs. |
| Template for Intervention Description and Replication (TIDieR) | Hoffmann et al. (2014) | Not a measurement tool to assess the external validity of RCTs. |
| TRANSFER Approach | Munthe-Kaas et al. (2020) | A comprehensive framework on steps/stages relevant to the assessment of external validity is presented. However, no specific measurement tool is presented, and the assessment with the TRANSFER approach is not suitable for reviews that do not include/perform a meta-analysis. |
| Treggiari´s external validity assessment | Treggiari et al. (2003) | Tool only applicable for studies on prevention of delayed ischemic neurological deficits with hypertension, hypervolemia, and hemodilution therapy following subarachnoid hemorrhage. |
| Van As´ Risk of bias assessment criteria for observational studies | Van As (2016) | Not a measurement tool to assess the external validity of RCTs. Tool not developed for RCTs. (RCTs were treated as prospective cohort studies in the review.) |
| Validity, Importance, Applicability, GReatness of benefit and Acceptability (VIAGRA) | Carroll (2020) | Not a measurement tool to assess the external validity of RCTs. |
| Quality of psychological trials for pain Scale | Yates et al. (2005) | Not a measurement tool to assess the external validity of RCTs. |
| **Excluded reports (Phase 2)** | | |
| **Title/Authors** | | **Reasons for exclusion** |
| “Mindfulness for pain, depression, anxiety, and quality of life in people with spinal cord injury: A systematic review” (Hearn & Cross, 2020) | | No data of measurement properties was calculated/evaluated for the “Selection bias”-dimension of the EPHPP tool individually. |
| “The TRANSFER Approach for assessing the transferability of systematic review findings” (Munthe-Kaas et al. 2020) | | No data of measurement properties was calculated/evaluated for the “indirectness”-dimension of the GRADE method. Furthermore, TRANSFER approach was excluded (see above). |
| “Is the Downs and Black scale a better tool to appraise the quality of the studies using virtual rehabilitation for post-stroke upper limb rehabilitation?” (Subramanian et al. 2019) | | No data of measurement properties was calculated/evaluated for the “External validity”-dimension of the Downs & Black-checklist individually. |
| “Depression in carers of people with dementia from a minority ethnic background: Systematic review and meta-analysis of randomised controlled trials of psychosocial interventions” (Akarsu et al., 2019) | | No data of measurement properties was calculated/evaluated for the “Selection bias”-dimension of the EPHPP tool individually. |
| “Do coursework summative assessments predict clinical performance? A systematic review” (Terry et al., 2017) | | No data of measurement properties was calculated/evaluated for the “External validity”-dimension of the Downs & Black-checklist individually. |
| “Efficacy and external validity of electronic and mobile phone-based interventions promoting vegetable intake in young adults: Systematic review and meta-analysis” (Nour et al., 2016) | | No evaluation of measurement properties of included tool was performed. |
| “Evidence quality in clinical guidelines: A comparison of two methods” (Cooper et al., 2015) | | No data of measurement properties was calculated/evaluated for the “indirectness”-dimension of the GRADE method individually. |
| “The relationship between external and internal validity of randomized controlled trials: A sample of hypertension trials from China” (Zhang et al., 2015) | | No evaluation of measurement properties of included tool was performed. |
| “Use of the functional movement screen in a tactical population: A review” (Bock et al., 2015) | | No data of measurement properties was calculated/evaluated for the “External validity”-dimension of the Downs & Black-checklist individually. |
| “Adding a "GRADE" to the quality appraisal of rheumatoid arthritis guidelines identifies limitations beyond AGREE-II” (Hazlewood et al., 2014) | | No data of measurement properties was calculated/evaluated for the “indirectness”-dimension of the GRADE method individually. |
| “Judging the quality of evidence in reviews of prognostic factor research: Adapting the GRADE framework” (Huguet et al., 2013) | | No evaluation of measurement properties of included tool was performed. |
| “A practical educational tool for teaching child-care hospital professionals attending evidence-based practice courses for continuing medical education to appraise internal validity in systematic reviews” (Rosati et al., 2012) | | No evaluation of measurement properties of included tool was performed. |
| “A comparison of the PEDro and Downs and Black quality assessment tools using the acquired brain injury intervention literature” (Aubut et al., 2013) | | No data of measurement properties was calculated/evaluated for the “External validity”-dimension of the Downs & Black-checklist individually. |
| “Prospective non-randomized studies in orthopaedics and traumatology: Systematic assessment of its methodological quality” (Pignataro et al., 2013) | | No data of measurement properties was calculated/evaluated for the “External validity”-dimension of the Downs & Black-checklist individually. |
| “External validity in healthy public policy: Application of the RE-AIM tool to the field of housing improvement” (Thomson & Thomas, 2012) | | No evaluation of measurement properties of included tool was performed. |
| “Interrater reliability and convergent validity of the American Academy for Cerebral Palsy and Developmental Medicine methodology for conducting systematic reviews” (Wiart et al., 2012) | | No data of measurement properties was calculated/evaluated for the “Selection bias”-dimension of the EPHPP tool individually. |
| “FORM: An Australian method for formulating and grading recommendations in evidence-based clinical guidelines” (Hillier et al., 2011) | | No evaluation of measurement properties of included tool was performed. |
| “The Australian 'FORM' approach to guideline development: The quest for the perfect system” (Dahm & Djulbegovic, 2011) | | No evaluation of measurement properties of included tool was performed. |
| “Can the Critical Appraisal Skills Programme check‐lists be used alongside Grading of Recommendations Assessment, Development and Evaluation to improve transparency and decision‐making?” (Purssell, 2020) | | No evaluation of measurement properties of included tool was performed. |
| “The use of Bayesian networks to assess the quality of evidence from research synthesis: 1” (Stewart et al., 2015) | | No evaluation of measurement properties of included tool was performed. |
| “The skills and experience of GRADE methodologists can be assessed with a simple tool.” (Norris et al., 2016) | | No data of measurement properties was calculated/evaluated for the “indirectness”-dimension of the GRADE method individually. |
| “The methodological quality assessment tools for preclinical and clinical studies, systematic review and meta-analysis, and clinical practice guideline: a systematic review.” (Zeng et al., 2015) | | Focused on internal validity only. |
| “Rating of Included Trials on the Efficacy–Effectiveness Spectrum: development of a new tool for systematic reviews” (Wieland et al., 2017) | | Already included in Phase 1. |
| “A checklist designed to aid consistency and reproducibility of GRADE assessments: Development and pilot validation” (Meader et al., 2014) | | Already included in Phase 1. |
| “How to assess the external validity and model validity of therapeutic trials: A conceptual approach to systematic review methodology” (Khorsan & Crawford, 2014) | | Already included in Phase 1. |
| “Examination of external validity in randomized controlled trials for adjuvant treatment of pancreatic adenocarcinoma” (Sorg et al., 2009) | | Already included in Phase 1. |
| “Chiropractic care for nonmusculoskeletal conditions: A systematic review with implications for whole systems research” (Hawk et al., 2007) | | Already included in Phase 1. |
| “Checklist for the qualitative evaluation of clinical studies with particular focus on external validity and model validity” (Bornhöft et al., 2006) | | Already included in Phase 1. |
| “A process for systematically reviewing the literature: Providing the research evidence for public health nursing interventions” (Thomas et al., 2004) | | Already included in Phase 1. |
| “Assessment of generalizability, applicability and predictability (GAP) for evaluating external validity in studies of universal family-based prevention of alcohol misuse in young people: systematic methodological review of randomized controlled trials.” (Fernandez-Hermida et al., 2012) | | Already included in Phase 1. |
| “A simple and valid tool distinguished efficacy from effectiveness studies.” (Gartlehner et al., 2006a) | | Already included in Phase 1. |
| “Criteria for Distinguishing Effectiveness From Efficacy Trials in Systematic Reviews” (Gartlehner et al., 2006b) | | No (additional) evaluation of measurement properties of included tool was performed. Data on measurement properties is the same as in the report above from Gartlehner et al. (2006a). |
| “Evaluating the relevance, generalization, and applicability of research: issues in external validation and translation methodology.” (Green & Glasgow, 2006) | | Already included in Phase 1. |
| “Instruments for assessing the quality of drug studies published in the medical literature.” (Cho & Bero, 1994) | | Already included in Phase 1. |
| “A quality assessment of randomized control trials of primary treatment of breast cancer.” (Liberati et al. 1986) | | Already included in Phase 1. |
| “The GRADE approach is reproducible in assessing the quality of evidence of quantitative evidence syntheses.” (Mustafa et al., 2013) | | No data of measurement properties was calculated/evaluated for the “indirectness”-dimension of the GRADE method individually. |
| “From the Trenches: A Cross-Sectional Study Applying the GRADE Tool in Systematic Reviews of Healthcare Interventions.” (Hartling et al., 2012) | | Percentage agreement was calculated for the indirectness domain, but authors did not use all items described in the GRADE handbook for indirectness. |
| Abbreviations: EPHPP = Effective Public Health Practice Project; GRADE = Grading of Recommendations Assessment, Development and Evaluation; RCT = randomized controlled trial, | | |
