# Supplementary material for: Identification of tools used to assess the external validity of randomized controlled trials in reviews: a systematic review of measurement properties
Source: BMC Med Res Methodol. 2022 Apr 6;22:100. doi: 10.1186/s12874-022-01561-5 (PMC8985274; doi:10.1186/s12874-022-01561-5)
Supplement: Supplementary file 4 — Additional file 4. [file 12874_2022_1561_MOESM4_ESM.docx]

**Identification of tools used to assess the external validity of randomized controlled trials in reviews: A systematic review of measurement properties**

Andres Jung, Julia Balzer, Tobias Braun & Kerstin Luedtke

**Table S4: Criteria for good measurement properties (CGMP) and GRADE rating results in detail**

Name of tool

***Measurement property* Certainty of evidence CGMP**

1. LEGEND (Clark et al., 2009)

***Content validity***: Starting point: MODERATE

-1 (serious risk of bias): **LOW (?)**

1. Carr´s Aplicability (Carr et al., 2004)

***Content validity***: Starting point: MODERATE

-2 (very serious risk of bias): **VERY LOW (?)**

1. Bornhöft´s checklist (Bornhöft et al., 2006)

***Content validity***: Starting point: MODERATE

-2 (very serious risk of bias): **VERY LOW (?)**

1. Clegg´s external validity assessment (Clegg et al., 2001)

***Content validity***: Starting point: MODERATE

-2 (very serious risk of bias)

-1 (serious indirectness: tool only applicable for England and Wales): **VERY LOW (?)**

1. Clinical Applicability (Haraldsson et al., 2006)

***Content validity***: Starting point: MODERATE

-2 (very serious risk of bias): **VERY LOW (?)**

1. Clinical Relevance Instrument (Cho & Bero, 1994)

***Content validity***: Starting point: HIGH

-1 (serious risk of bias): **MODERATE (?)^1^**

^1^ Content validation was not performed for the final version. Furthermore, it is unclear if “relevance” and comprehensiveness” were assessed for content validity.

***Reliability:***

-1 (serious risk of bias) **MODERATE (-)**

1. Clinical Relevance of CCBRG (Van Tulder et al., 2003)

***Content validity***: Starting point: High

-1 (serious risk of bias): **MODERATE (?)**

1. Clinical relevance scores (Karjalainen et al., 2000)

***Content validity***: Starting point: MODERATE

-2 (very serious risk of bias): **VERY LOW (?)**

1. Estrada´s applicability assessment criteria (Estrada et al., 2021)

***Content validity*:** Starting point: MODERATE

-1 (serious risk of bias):

-1 (serious indirectness*): **VERY** **LOW (?)**

* *Tool only applicable for Latin America*

1. EVAT (Khorsan & Crawford, 2014)

***Content validity*:** Starting point: MODERATE

-1 (serious risk of bias): **LOW (?)**

1. External Validity of the Downs & Black- checklist (Downs & Black, 1998)

***Content validity***: Starting point: HIGH

-1 (serious risk of bias): **MODERATE (?)^2^**

^2^ Face and content validation was performed for the pilot version. However, there is no evidence or information, whether the final version was assessed for face or content validity as well.

***Internal consistency****:*

-2 (very serious risk of bias)

-2 (imprecision, n<50): **VERY LOW (?)**

***Reliability****:*

-1 (imprecision, n=50-100) **MODERATE (±)^3^**

^3^ **(-)** *in Downs and Black (1998) (inexperienced raters) and* **(+)** *in O´Connor et al. (2015)(experienced raters)*

***Measurement error:***

-2 (very serious risk of bias)

-2 (imprecision, n<50) **VERY LOW (?)**

***Construct validity****:*

-1 (serious risk of bias)

-2 (imprecision, n<50): **VERY LOW (-)^4^**

**^4^** Results not in accordance with the hypothesis

1. External validity of Foy´s checklist (Foy et al., 2010)

***Content validity***: Starting point: MODERATE

-2 (very serious risk of bias): **VERY LOW (?)**

1. External validity of Liberati´s quality assessment criteria (Liberati et al., 1986)

***Content validity***: Starting point: MODERATE

-2 (very serious risk of bias): **VERY LOW (?)**

1. External validity of Sorg´s checklist (Sorg et al., 2009)

***Content validity***: Starting point: MODERATE

-2 (very serious risk of bias): **VERY LOW (?)**

1. External validity criteria of the USPSTF manual (2008; 2015)

***Content validity***: Starting point: MODERATE

-2 (very serious risk of bias)

-1 (serious indirectness: tool only applicable for U.S.): **VERY LOW (?)**

***Reliability:***

-2 (imprecision, n<50)

-1 (serious indirectness: tool only applicable for U.S.): **VERY** **LOW (+)**

1. FAME (Averis & Pearson , 2003)

***Content validity***: Starting point: MODERATE

-2 (very serious risk of bias): **VERY LOW (?)**

1. GAP (Fernandez-Hermida et al., 2012)

***Content validity***: Starting point: MODERATE

-2 (very serious risk of bias): **VERY LOW (?)**

1. Gartlehner´s tool (Gartlehner et al., 2006)

***Content validity***: Starting point: MODERATE

-2 (very serious risk of bias)

-1 (serious indirectness*): **VERY LOW (?)**

***Reliability****:*

-1 (serious indirectness*): **MODERATE (-)**

***Measurement error:***

-1 (serious risk of bias)

-2 (imprecision, n<50)

-1 (serious indirectness*) **VERY LOW (?)**

***Criterion validity****:*

-1 (serious risk of bias)

-2 (imprecision, n<50)

-1 (serious indirectness*): **VERY LOW (+)**

* *the construct the tool aims to measure, is indirectly related to the external validity of RCTs. It is designed to distinguish between efficacy and effectiveness studies based on criteria on study design.*

1. Green & Glasgow´s external validity rating criteria (Green & Glasgow, 2006)

***Content validity***: Starting point: MODERATE

-2 (very serious risk of bias):

-1 (serious indirectness*): **VERY LOW (?)**

***Reliability:***

-1 (serious risk of bias)

-2 (imprecision, n<50)

-1 (serious indirectness*): **VERY LOW (+)**

***Construct validity****:*

-1 (serious risk of bias)

-2 (imprecision, n<50)

-1 (serious indirectness*): **VERY LOW (-)^5^**

* *the construct the tool aims to measure, is indirectly related to the external validity of RCTs. It is designed to measure the report quality on factors relevant for the external validity, but not the external validity itself.*

^5^ Results not in accordance with the hypotheses

1. “Indirectness” from the GRADE handbook (Schünemann et al., 2013)

***Content validity***: Starting point: MODERATE

**MODERATE (?)**

***Reliability:***

-3 (extremely serious risk of bias): **VERY LOW (-)**

1. Loyka´s external validity framework (Loyka et al., 2020)

***Content validity***: Starting point: MODERATE

-1 (serious risk of bias)

-1 (serious indirectness*): **VERY** **LOW (?)**

***Reliability:***

-1 (serious risk of bias)

-1 (serious indirectness*): **LOW (?)**

** tool developed for generalizability of research in psychological science*

1. Modified indirectness of the Checklist for GRADE (Meader et al., 2014)

***Content validity***: Starting point: MODERATE

-1 (serious indirectness*): **LOW (?)**

***Reliability:***

-1 (serious risk of bias)

-2 (imprecision, n<50)

-1 (serious indirectness*): **VERY LOW (-)**

* *the construct the tool aims to measure, is indirectly related to the external validity of RCTs. It is designed to assess the applicability of meta-analyses of RCTs, but the applicability of individual RCTs is not rated.*

1. NHMRC Handbook (2001)

***Content validity***: Starting point: MODERATE

-2 (very serious risk of bias): **VERY LOW (?)**

1. Revised GATE (NICE Guideline, 2012)

***Content validity*:** Starting point: MODERATE

-2 (very serious risk of bias): **VERY LOW (?)**

1. RITES (Wieland et al., 2017)

***Content validity****:* Starting Point: HIGH

-1 (serious indirectness*): **MODERATE (+)**

***Reliability:***

-3 (extremely serious risk of bias)

-2 (imprecision, n<50)

-1 (serious inconsistency)^6^

-1 (serious indirectness*): **VERY LOW (+)^6^**

**^6^** the results were inconsistent within the reliability study by Aves (2017). According to COSMIN, if the majority of the results are sufficient, an overall rating of “sufficient” can be considered with downgrading the quality of the evidence for inconsistency.

***Construct validity****:*

-1 (imprecision, n=50-100)

-1 (serious indirectness*): **LOW (+)**

* *the construct the tool aims to measure, is indirectly related to the external validity of RCTs. It is designed to characterize the efficacy-effectiveness nature of trials included in systematic reviews.*

1. “Selection Bias”-dimension (Section A) of EPHPP (Thomas et al., 2004)

***Content validity****:* Starting Point: HIGH

-1 (serious risk of bias): **MODERATE (?)^7^**

^7^ It is assumable that the content validation was performed for the final version of the tool. But with only two items to assess the external validity of clinical trials, a sufficient (+) rating could not be given, because of doubtful comprehensiveness. Furthermore, it is not clear if content validation was performed for the individual dimension of interest.

***Reliability****:*

-1 (serious risk of bias)

-1 (imprecision, n=50-100): **LOW (-)**

***Construct validity****:*

-2 (very serious risk of bias)

-1 (imprecision, n=50-100): **VERY LOW (+)**

1. Section D of the CASP checklist for RCTs (Critical Appraisal Skills Programme, 2020)

***Content validity***: Starting point: MODERATE

-2 (very serious risk of bias): **VERY LOW (?)**

1. Whole Systems research considerations´checklist (Hawk et al., 2007)

***Content validity***: Starting point: MODERATE

-2 (very serious risk of bias): **VERY LOW (?)**

**Appendix:**

**COSMIN´s modified GRADE approach on content validity taken from Terwee et al., (2018)**

| **Study design** | **Quality of evidence** | **Lower if** |
| --- | --- | --- |
| At least 1 content validity study  No content validity studies | High  Moderate  Low  Very low | Risk of bias  -1 Serious  -2 Very serious  Inconsistency  -1 Serious  -2 Very serious  Indirecntess  -1 Serious  -2 Very serious |
| The level of evidence indicates how confident we are that the overall ratings are trustworthy. The starting point is the assumption that the evidence is of high quality. The quality of evidence is subsequently downgraded with one or two levels per factor to moderate, low, or very low when there is risk of bias (low study quality), (unexplained) inconsistency in results, or indirect results | | |

**COSMIN´s modified GRADE approach (for all measurement properties except content validity) taken from Prinsen et al., (2018)**

| **Quality of evidence** | **Lower if** |
| --- | --- |
| High  Moderate  Low  Very low | Risk of bias  -1 Serious  -2 Very serious  -3 Extremely serious  Inconsistency  -1 Serious  -2 Very serious  Imprecision  -1 total n = 50-100  -2 total n < 50  Indirecntess  -1 Serious  -2 Very serious |
| The starting point is the assumption that the evidence is of high quality. The quality of evidence is subsequently downgraded with 1-3 levels for each factor (i.e., risk of bias, inconsistency, imprecision, indirectness) to moderate, low, or very low when there is risk of bias (low study quality), (unexplained) inconsistency in results, or indirect results. Information on how to downgrade is described in detail in the COSMIN user manual (http://www.cosmi  n.nl/)  n = sample size | |

Both tables are from research published under the terms of the Creative Commons Attribution 4.0 International License (http://creativecommons.org/licenses/by/4.0/), which permits unrestricted use, distribution, and reproduction in any medium, provided appropriate credit to the original author(s) and the source is given.

No changes were made by the review authors.

Prinsen, C. A., Mokkink, L. B., Bouter, L. M., Alonso, J., Patrick, D. L., De Vet, H. C., & Terwee, C. B. (2018). COSMIN guideline for systematic reviews of patient-reported outcome measures. *Quality of Life Research*, *27*(5), 1147-1157.

Terwee, C. B., Prinsen, C. A., Chiarotto, A., Westerman, M. J., Patrick, D. L., Alonso, J., ... & Mokkink, L. B. (2018). COSMIN methodology for evaluating the content validity of patient-reported outcome measures: a Delphi study. *Quality of Life Research*, *27*(5), 1159-1170.
